# Mechanisms of Carrageenan-Induced Immune Inflammation and Thrombosis: A Narrative Review

**DOI:** 10.3390/foods15203727

**Published:** 2026-10-19

**Authors:** Xiaomu Ma, Daihan Wu, Ren Huang

**Affiliations:** 1 Department of Clinical Medicine, Shantou University Medical College, Shantou 515041, China; joy_xiaomuma@outlook.com (X.M.);; 2 Laboratory Animal Center, Shantou University Medical College, Shantou 515041, China

**Keywords:** carrageenan, evidence stratification framework, sulfate groups, immune inflammation, thrombosis, toll-like receptors, NLRP3 inflammasome, NETs, CLEC-2

## Abstract

This review summarizes carrageenan (CGN)-induced immune inflammation and thrombosis, route-dependent effects, and studies conducted in animal models, distinguishing experimental conditions from human dietary exposure. PubMed, Web of Science, and CNKI were searched, and studies were stratified by subtype, dose, route, and model. Toll-like receptor 4 (TLR4) is central to CGN-mediated immune activation, but the CGN–TLR4/MD-2 co-crystal structure remains undetermined. MyD88 mediates TNF-α, and TRIF-dependent NLRP3 contributes to IL-1β maturation, but this dual-branch pathway has been confirmed in mouse peritoneal macrophages only and does not activate IRF3/IFN-β. Route determines outcomes: intravenous CGN activates FXII/CLEC-2, causing rapid thrombosis; intraperitoneal CGN triggers macrophage TNF-α/IL-1β release, initiating the inflammation–endothelial injury–coagulation cascade; and oral CGN has been shown under specific experimental model conditions to be degraded by gut microbiota into low-molecular-weight fragments, which may potentially induce colitis. These outcomes have not been confirmed to be the consequences of typical human dietary exposure. These findings were primarily derived from non-oral, high-dose, degraded-product, or in vitro studies and cannot be directly extrapolated to human dietary exposure. Food-grade CGN, low-molecular-weight/degraded CGN, and poligeenan differ fundamentally in metabolism and toxicology. Under conditions of an intact barrier and normal dietary exposure, the current evidence does not indicate a consistent pro-inflammatory or pro-thrombotic risk of food-grade CGN; however, this does not exclude all potential effects, as microbiota degradation efficiency, fragment exposure, and special-population susceptibility have not been fully investigated. Future studies should determine the CGN–TLR4/MD-2 structure, validate the dual-branch pathway independently, and conduct human metabolic tracing and special-population studies.

## 1. Introduction and Research Methods

### 1.1. Background and Current Controversies

Carrageenan (CGN) is a sulfated linear galactan obtained from marine red algae of the genera *Eucheuma* and *Chondrus* in the phylum Rhodophyta via alkali extraction and purification [1,2]. In 1819, Dawson Turner observed that this red alga forms a gel after boiling and cooling [3]. Since the development of the seaweed processing industry, CGN has been used as a thickening agent, stabilizer, and gelling agent in food products (such as jelly and ice cream) and everyday chemical products [4,5,6]. CGN has also demonstrated significant translational value in the pharmaceutical field as an excipient, drug carrier, and biomaterial matrix [5,7,8]. Since the 1950s, researchers have reported that high-dose crude CGN extracts can induce intestinal inflammation in animals [9,10,11,12]. In 1962, Winter et al. first established the CGN rat paw edema model, which became a classic method for the in vivo screening of anti-inflammatory drugs [13]. In 1973, Giroud et al. first reported CGN-induced thrombosis [14]. Since the 1980s, separation techniques for high-purity CGN subtypes have matured, making it possible to study the relationships between molecular weight, structural subtypes, and biological activities.

However, disagreements regarding the safety of CGN persist, with contradictory research data and conclusions regarding its biological activities and mechanisms. In 2019, McKim et al. [15] clearly distinguished food-grade CGN from poligeenan (PGN). They noted that food-grade CGN (typically ≥100 kDa) fundamentally differs from poligeenan (PGN, 10–20 kDa, an industrial acid hydrolysis product) and partially degraded low-molecular-weight CGN/degraded CGN (LMW-CGN/dCGN, 20–40 kDa) in chemical properties, in vivo metabolism, and toxicological profile. Furthermore, CGN is not converted into PGN in vivo, supporting its safety for food applications. Kimilu et al. [16] reported that PGN may induce intestinal damage, but the risk of food-grade CGN for patients with inflammatory bowel disease (IBD) remains unclear. Komisarska [17] and Borsani [18] reported that CGN can disrupt the intestinal microenvironment by activating the TLR4/NF-κB pathway and may mediate allergies and intestinal inflammation, respectively. Tobacman [19] proposed that CGN exposure is associated with intestinal lesions, but Weiner [20] pointed out that the materials used in the supporting studies were mostly non-food-grade and of indeterminate purity, rendering the relevant conclusions unreliable.

The above reviews summarized the biological activities of CGN from the perspectives of oral toxicology, the gut microbiome, inflammatory bowel disease, and allergic reactions [15,16,17,18,19,20], but they have shortcomings. First, there is a lack of systematic integration of the coupling mechanism between inflammation and thrombosis. Although some reviews [17,18] described the TLR4/NF-κB pathway, they did not extend this mechanistic framework to the vascular consequences of sustained NF-κB activation, such as upregulated tissue factor (TF) expression, elevated expression of endothelial adhesion molecules, and disruption of vascular homeostasis. Second, the influential role of administration route has received little attention. CGN administered via intravenous injection, intraperitoneal injection, and oral routes triggers distinct effect patterns, and no review has yet systematically classified and elaborated these mechanistic differences by administration route. Third, the hierarchy of the evidence reported in this literature is unclear. Existing reviews often conflate data from high-concentration in vitro experiments, high-dose animal model exposures, non-oral administration routes, and normal human dietary exposure, leading to overgeneralization of safety hazard conclusions. Furthermore, there are very few reviews on CGN-induced thrombosis, and there is a lack of studies that systematically integrate the “immune inflammation–vascular endothelial injury–coagulation activation” pathway as a complete cascade.

### 1.2. Purpose and Novelty of This Review

This review was conducted to answer three questions: (1) Through which molecular pathways does CGN initiate immune inflammation, and how is it coupled with endothelial injury and coagulation activation? (2) How does the route of administration determine the effect pattern of CGN? (3) Which conclusions are applicable to human conventional dietary exposure, and which are applicable only to specific experimental conditions? To answer these questions, immune inflammation initiation (TLR4/NF-κB/NLRP3), vascular endothelial injury (oxidative stress, adhesion molecules, and neutrophil extracellular traps), and coagulation activation (FXII/CLEC-2/thrombin generation) are incorporated into a unified analytical framework and compared by intravenous, intraperitoneal, oral, and subcutaneous/local routes of administration. Furthermore, the evidence is stratified by CGN subtype, molecular weight, dose, and model system. Compared with previous reviews, this article’s novelty lies in the construction of an “inflammation–thrombosis” coupled network by clearly stratifying the evidence from the included literature according to a hierarchy based on the principle of similarity to human dietary exposure; systematically emphasizing the decisive role of the route of administration; and clearly distinguishing evidence of the safety of food-grade CGN under conventional dietary exposure from mechanistic findings under specific experimental conditions, avoiding direct extrapolation of the latter to food additive safety assessment.

### 1.3. Type of Study, Literature Search Methodology and Three-Tier Evidence Stratification Framework

Given the substantial heterogeneity between the included studies regarding experimental design, detection endpoints, CGN subtypes/molecular weights, doses, and model systems, a systematic review or meta-analysis was not feasible. Therefore, a narrative synthesis approach was adopted to integrate multi-line evidence ranging from in vitro mechanistic studies to animal models.

The databases searched included PubMed, Web of Science, and CNKI, with the search period starting from the inception of each database to 30 April 2026. No language restrictions were applied. The main search terms included “carrageenan,” “poligeenan,” “degraded carrageenan,” “TLR4,” “inflammation,” “thrombosis,” “endothelial dysfunction,” “gut microbiota,” and “intestinal permeability,” along with their synonyms and Medical Subject Headings (MeSH) terms. Inclusion criteria were research articles, reviews, or reports from authoritative institutions that assessed the effects of different CGN subtypes or molecular weight fractions on inflammation, immune regulation, gut microbiota, intestinal barrier function, thrombosis, or endothelial function. Exclusion criteria included conference abstracts, inability to extract key experimental conditions, studies unrelated to CGN, duplicate publications, those involving only products/patents, and non-peer-reviewed materials.

Literature screening was conducted with an initial review of titles and abstracts, followed by full-text reviews. Two authors independently screened the literature, resolving disagreements through discussion. Key data, including CGN subtype, molecular weight, dose, administration route, model system, and main findings, were manually extracted.

Existing research findings on carrageenan-induced inflammation and thrombosis come from diverse experimental conditions, including in vitro mechanistic assays, high-dose non-oral-administration animal models, and high-dose oral-administration animal studies; there are limited human-related data. To clearly distinguish the translational reference value of various types of evidence for the human context and to avoid over-extrapolating non-clinical mechanistic findings, we propose an author-defined three-tier evidence-stratification conceptual framework for organizing heterogeneous evidence in narrative reviews. This framework primarily categorizes the existing evidence based on two core dimensions: (1) the similarity between experimental exposure conditions and real-world human dietary exposure to food-grade carrageenan and (2) the degree of translational reference of each experimental system for inferring human safety. This framework distinguishes non-clinical evidence used for mechanistic exploration from evidence with greater reference value for human dietary exposure, enabling readers to clearly discern which findings represent mechanistic observations for hypothesis generation and which findings are more suitable for deriving conclusions related to human dietary exposure.

Category A comprises studies using food-grade (≥100 kDa) CGN, administered orally, at doses physiologically relevant to human dietary exposure, which are directly applicable for assessing human dietary exposure relevance. Category B comprises studies using non-oral routes, high doses, or those designed to establish animal models, which are suitable for understanding pharmacological/toxicological mechanisms and efficacy screening but whose findings are not directly extrapolable to food additive safety evaluation. Finally, Category C comprises studies based primarily on in vitro cell experiments or those using non-food-grade PGN/LMW fragments, intended to propose molecular mechanism hypotheses that still require validation under in vivo oral conditions.

In this narrative synthesis, Category A evidence was prioritized for conclusions related to human dietary exposure, Category B evidence was used for mechanistic chain and animal model interpretation, and Category C evidence was employed for mechanistic hypotheses and structure–activity relationship discussions. When conclusions from different levels of evidence were inconsistent, the source, experimental conditions, and extrapolation limitations were explicitly noted. Key experimental evidence is summarized in Table 1 according to the aforementioned dimensions.

## 2. Chemical Structure, Subtypes, and Nomenclature by Molecular Weight Fractions

### 2.1. Basic Chemical Structure and Molecular Weight

Natural CGN comprises a class of high-molecular-weight sulfated galactans, wherein the core structure of κ- and ι-subtypes consists of alternating β-D-galactose and 3,6-anhydro-α-D-galactose linked by β-1,4 and α-1,3 glycosidic bonds, forming regular repeating disaccharide units. λ-carrageenan lacks 3,6-anhydro-α-D-galactose units [55,56]. Its molecular weight range is typically 100–1000 kDa [57,58] and is influenced by seaweed species, growth environment, and extraction processes (temperature, pH, and degree of acid–base degradation) [57,58,59]. Critical gelation molecular weights differ between CGN subtypes: κ-type and ι-type are 130–210 and 280 kDa, respectively, while λ-type lacks gelation capacity. According to JECFA 2014 standards, the weight-average molecular weight (Mw) of food-grade CGN must exceed 100 kDa, and commercial high-quality, food-grade CGN typically has a molecular weight in the range of 200–800 kDa [60]. In contrast, poligeenan (PGN), an industrial degradation product of CGN, has a molecular weight of only 10–20 kDa, is classified as non-food-grade, and exhibits strong pro-inflammatory bioactivity [60].

### 2.2. Nomenclature of CGN Based on Molecular Weight Fractions

From the 1950s to the 1970s, the academic and industrial sectors classified CGN subtypes solely based on chemical configuration, without distinguishing molecular weight differences, grouping fragments of varying molecular weights and considering them uniformly as CGN. This led to contradictory conclusions in toxicological and safety studies. Subsequent research confirmed that the biosafety of high-molecular-weight natural CGN and that of low-molecular-weight degraded CGN are fundamentally different, which is the core reason for the conflicting conclusions.

From 1969 to 1971, multiple studies found that the oral administration of high doses of non-food-grade, acid-hydrolyzed, degraded CGN were capable of inducing intestinal inflammation. Marcus and Watt first confirmed in 1969 that oral administration of degraded CGN induced colitis in guinea pigs [61], and in 1971, they further clarified the pathological features of colonic ulcers [62], laying the foundation for animal models of inflammatory bowel disease. The academic community gradually recognized that low-molecular-weight fragments generated from the degradation of natural CGN are crucial to its increased toxicity. Terms such as low-molecular-weight CGN (LMW-CGN), degraded CGN (d-CGN), and PGN emerged successively. However, early studies commonly confused these three substances, leading to public misconceptions about food-grade CGN and prompting the industry to establish a molecular-weight-based nomenclature standard.

In 1976, Pittman et al. divided d-CGN into two fractions, 20–40 and 10–20 kDa, confirming differences in their functions and toxicity, thereby laying the foundation for the nomenclature and mechanism studies on PGN [63]. McKim et al. further clarified that PGN corresponds to ultra-low-molecular-weight degradation products of 10–20 kDa, 20–40 kDa corresponds to moderately degraded d-CGN, and food-grade natural CGN is ≥100 kDa [15]. In a study using peritoneal macrophages, they also observed that mammals lack endo-α-1,3-galactosidase and cannot stably generate CGN degradation fragments <50 kDa [15]. In 2018, the EFSA released a re-evaluation report, officially confirming that PGN is a non-food-grade harmful degradation product whose addition to food is prohibited, resolving 40 years of nomenclature confusion.

Molecular weight is one of the important determinants of CGN function and safety. PGN of 10–20 kDa has the strongest ability to penetrate the peritoneal and intestinal barriers and the highest pro-inflammatory toxicity [16]. It can only be generated through the use of strong industrial acids and high temperatures [64], with no corresponding degradation enzyme present in humans or animals [15]. LMW-CGN/d-CGN of 20–40 kDa has weaker penetrability and pro-inflammatory toxicity and can originate from industrial degradation or metabolism by the human gut microbiota [52,65]. It is challenging for natural food-grade CGN ≥100 kDa to penetrate the intestinal epithelium under conditions of an intact intestinal barrier, and no significant toxicity was observed with dietary-level oral exposure [60,66,67,68]. However, in cases of high doses (>5% feed addition) or models with an impaired intestinal barrier, some studies reported mild inflammatory responses [16,17,48], indicating that the safety of food-grade CGN is dose- and intestinal barrier-status-dependent.

In the “substance nomenclature”, LMW-CGN/d-CGN traditionally refers to non-food-grade products obtained through partial degradation under industrial or experimental conditions, such as high heat, oxidation, and extremely alkaline/weakly acidic environments. It has a molecular weight of approximately 20–40 kDa and is not used as a food additive. However, gut microbial activity (e.g., *Bacteroides xylanisolvens*) can induce food-grade CGN to produce small quantities of low-molecular-weight fragments in situ, and these fragments may exhibit pro-inflammatory activity [52]. Therefore, it is necessary to distinguish between two sources: industrially or experimentally prepared LMW-CGN/d-CGN, which is a non-food-grade substance, and trace low-molecular-weight fragments generated in situ from food-grade CGN by gut microbial activity, whose yield, molecular weight distribution, exposure concentration, and duration may be far lower than those of industrially prepared LMW-CGN/d-CGN. The existing evidence is insufficient to determine whether fragments generated in situ in the human gut reach the 20–40 kDa range or accumulate to biologically significant concentrations. Thus, the statement that “food-grade CGN can generate trace LMW fragments in the gut” cannot be equated with “LMW-CGN/d-CGN is a food-grade substance”. Likewise, toxicological findings from industrial LMW-CGN/d-CGN cannot be directly extrapolated to food-grade CGN under normal dietary exposure conditions, even though trace LMW fragments may be produced via gut microbial metabolism.

A comparison of the three types of substances is shown in Table 2.

### 2.3. Subtype Classification and Structural Characteristics

The CGN subtype classification system has been progressively refined. In 1953, Smith et al. proposed the κ/λ binary classification system [70]; in their research performed from 1963 to 1965, the Rees team added the ι type, establishing a κ/ι/λ ternary core classification [71,72]. In 1971, IUPAC and in 2007, the FAO/JECFA successively standardized this system, forming a globally accepted classification standard [73]. Based on differences in sulfation degree, glycosidic bond configuration, sulfate group substitution position, and molecular conformation, CGN can be divided into seven subtypes: κ, ι, λ, γ, ν, ξ, and μ, among which κ, ι, and λ are the most extensively studied [1,56,60,69,74].

**Figure 1:**
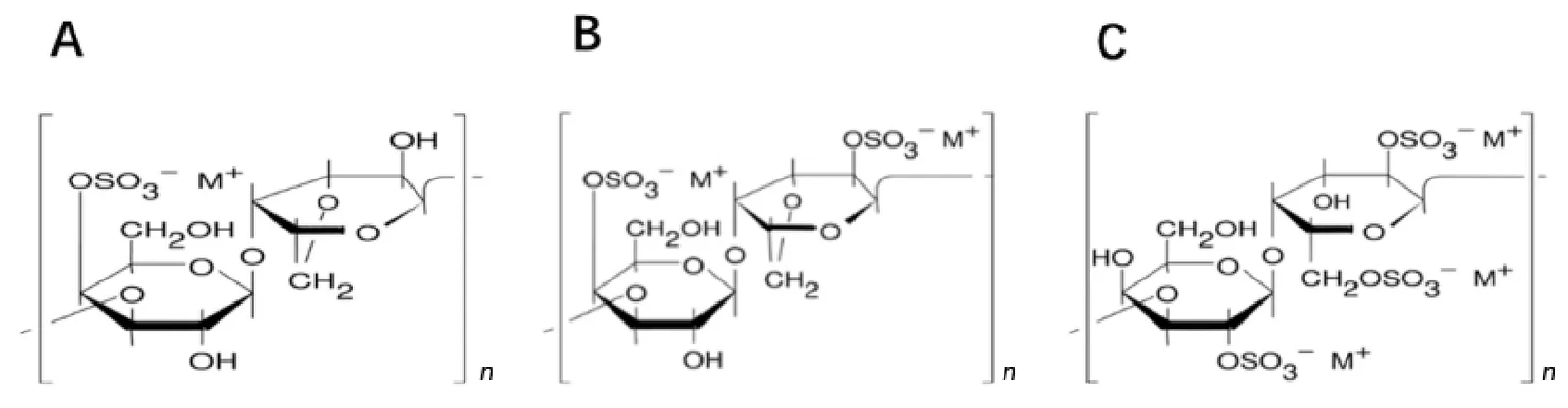
Molecular structures of the disaccharide units of three common carrageenan subtypes. (**A**) κ-carrageenan: The sulfate group is located at the C-4 position of 1,3-linked β-D-galactose (Gal-4-SO_3_^−^); (**B**) ι-carrageenan: Two sulfate groups are, respectively, located at the C-4 position of 1,3-linked β-D-galactose (Gal-4-SO_3_^−^) and the C-2 position of 3,6-anhydro-α-D-galactose (DA-2-SO_3_^−^); (**C**) λ-carrageenan: Its structure does not contain a 3,6-anhydro bridge, and three sulfate groups are, respectively, located at the C-2 position of 1,3-linked β-D-galactose (Gal-2-SO_3_^−^), as well as the C-2 and C-6 positions of 1,4-linked α-D-galactose (Gal-2-SO_3_^−^, Gal-6-SO_3_^−^).

κ-CGN: The disaccharide unit contains 3,6-anhydro-α-D-galactose (DA), forming an intramolecular ether bridge between C3 and C6, restricting the rotation of the sugar ring; it contains one sulfate group located at the C-4 position of β-D-galactose, with a sulfation mass fraction of 25–30%, the lowest among the three. ι-CGN: The disaccharide unit contains 3,6-anhydro-α-D-galactose (DA), forming an intramolecular ether bridge between C3 and C6, restricting the rotation of the sugar ring; it contains two sulfate groups, located at the C-4 position of β-D-galactose and the C-2 position of 3,6-anhydrogalactose, with a sulfation mass fraction of 28–35%. λ-CGN: It contains three sulfate groups, located at the C-2 position of β-D-galactose and the C-2 and C-6 positions of α-D-galactose; its 4-linked galactose residue is α-D-galactose instead of 3,6-anhydro-α-D-galactose, so λ-CGN lacks the 3,6-internal ether bridge structure. It has a sulfation mass fraction of 34–38%, the highest of the three [60]. The molecular structures of the three common subtypes κ, ι, and λ are shown in Figure 1.

### 2.4. Molecular Conformation and Functional Properties of Subtypes

The degree of sulfation and glycosidic bond configuration determine molecular conformation and function. κ-CGN can form a rigid double-helical polymer structure via intermolecular hydrogen bonds, with optimal gelation performance; ι-CGN has a relatively loose double helix, with gel strength weaker than κ-type but better elasticity; λ-CGN, due to the absence of internal ether structures, cannot form a helical structure, exists in a random coil state, and only has thickening properties [1,55,75]. Rheological studies confirm that the subtypes’ gelation ability ranks as follows: κ-CGN > ι-CGN > λ-CGN [58]. Sulfate groups impart a negative charge to the CGN molecular chain, generating intermolecular electrostatic repulsion that inhibits gel network formation [76], while cations such as K^+^, Ca^2+^, and NH_4_^+^ can shield the negative charge, promoting helical aggregation and gel stabilization [77]. Therefore, the greater the number of sulfate groups, the stronger the water solubility and the weaker the gelation ability [5,78].

Few systematic studies have directly compared the effects of different CGN subtypes on immune inflammation and thrombosis induction. Bhattacharyya et al. (2010) used human colonic epithelial NCM460 cells to compare the in vitro pro-inflammatory activities of κ-, ι-, and λ-carrageenans, using IL-8, BCL10, and ROS as evaluation indicators, and found that λ-CGN induced the strongest secretion of inflammatory cytokines, while ι-CGN was the weakest [49]. In 1981, Thomson et al. intraperitoneally injected 125 mg/kg of λ, κ, and ι-CGN into Sprague Dawley rats and found that all three subtypes could induce thrombocytopenia and coagulation disorders, with the order of potency being λ ≈ κ > ι [79]; in 1985, Bekemeier et al. further compared the thrombogenic abilities of the three subtypes and concluded that κ-CGN had the strongest thrombogenic activity [80]. The literature suggests that the correlation between subtypes, conformation, and function remains unclear and may be influenced by factors such as dosage, route of administration, model system, and detection endpoints, requiring further research.

## 3. Immune Inflammatory Activation Mechanism Centered on Toll-like Receptor 4

### 3.1. Molecular Basis of CGN Recognition of TLR4/MD-2: Experimental Evidence and Inferred Model

TLR4 is a transmembrane glycoprotein that is mainly expressed in myeloid cells, vascular endothelial cells, and intestinal epithelial cells. It serves as the pattern recognition receptor for lipopolysaccharide (LPS) and plays a key role in initiating innate immune responses [81]. Its ligand spectrum includes bacterial LPS and other pathogens, as well as specific sulfated polysaccharides (e.g., CGN) [48]. Based on studies of the LPS-TLR4/myeloid differentiation protein 2 (MD-2)’s co-crystal structure, TLR4 must form a complex with the accessory protein MD-2 to recognize ligands and initiate downstream signaling, inducing TLR4 dimerization and activation [81]. The specific binding mode of CGN with the TLR4/MD-2 complex has primarily been inferred through analogy with sulfated polysaccharides, and direct evidence from co-crystal structures is lacking.

Regarding the molecular epitopes involved in the recognition and binding of CGN to TLR4, scientific understanding has been progressively updated with advancements in structural biology and cellular functional experiments. In 2010, Bhattacharyya et al. [49] first proposed that the molecular basis for CGN binding to TLR4 is the α-1,3-galactoside epitope. Using human colonic epithelial NCM460 cells as a model, they found that pretreatment of CGN with α-1 → (3,6-anhydro)galactosidase significantly reduced the expression levels of IL-8 and BCL10 in cells; conversely, enzymatic disruption of the β-1,4 glycosidic bonds in κ-type and ι-type CGN led to the upregulation of inflammatory factors [49]. The researchers attributed this phenomenon to the exposure of cryptic α-1,3-galactoside immune epitopes within the long-chain CGN after enzymatic cleavage, which were then recognized by TLR4 and activated the inflammatory response.

However, Wahnou H argued that this study merely reported mechanistic speculation, as it lacked detailed structural characterization of CGN and had several limitations, including inadequate experimental design, inference of polysaccharide structure indirectly from enzyme function, and oversimplified mechanistic extrapolations [82].

Subsequent experiments gradually refuted the hypothesis of a single α-1,3-galactoside epitope. In 2015, McKim et al. [83], based on HEK293 TLR4 reporter cell models, found that food-grade CGN could not directly activate TLR4 homodimers. In 2019, Myers et al. [54], by combining TLR reporter cells with gene-silencing experiments, confirmed that the pro-inflammatory effects of λ-type CGN were primarily mediated by TLR2/6 heterodimers, secondarily by TLR4/6 heterodimer pathways, and did not activate the classical TLR4 homodimer signaling, suggesting that CGN’s receptor recognition preference is subtype structure-dependent.

In 2020, Lopes et al. [53], using primary peritoneal macrophages from TLR4 knockout and MyD88/TRIF-deficient mice for mechanistic validation experiments, clarified that the pro-inflammatory activity of low-molecular-weight κ-type CGN strictly depends on the TLR4 pathway, activating MyD88 and TRIF downstream signaling pathways. Additionally, Lopes et al. [53] conducted limulus amebocyte lysate (LAL) endotoxin detection in the experiments, confirming that the κ-type CGN used was free of endotoxin contamination, ruling out LPS-induced TLR4 activation. However, this study did not involve spatial conformational analysis of the TLR4 binding site.

Detailed glycochemical analysis [84] identified the true backbone structure of CGN, which completely lacks the Galα1 → 3Gal α-1,3-galactoside epitope mentioned by Bhattacharyya et al., directly overturning the single disaccharide epitope recognition mechanism they proposed [49] and providing direct structural evidence for establishing a novel recognition model for CGN and TLR receptors.

Based on chemical structural identification (FT-IR, elemental analysis, and ^1^H/^13^C NMR) of the carrageenan (CGN) and its derivatives, Liang et al. [8] evaluated the relationships between the molecular weight of red seaweed galactans (including κ-carrageenan and its oligosaccharides), sulfate group substitution position, and biological activities, using the rabbit whole blood method (APTT and PT tests) and the human umbilical vein endothelial cell (HUVEC) MTT assay. Their study showed that the anticoagulant activity of CGN depends primarily on the substitution position of sulfate groups rather than the degree of sulfate groups; sulfation at the C-2 position of 3,6-anhydro-α-D-galactose (A-2) and the C-6 position of β-D-galactose (G-6) is the most favorable. Molecular weight has only a partial effect on anticoagulant activity, and this effect is less pronounced than that of the substitution position. The proposed order of cytotoxicity for sulfate groups at different positions is G-6 > G-4 > G-2 > A-2. The authors suggest that the cytotoxicity of κ-carrageenan, on the basis of its primary structure, may arise from the double-helix secondary structure formed through the oriented association of sulfate groups and hydrogen bonds. The distribution characteristics of sulfate groups in the secondary structure of the polymeric chain may play a key role in the interaction between the cell surface and glycans.

Given this information, a novel composite epitope hypothesis for CGN recognition by TLRs can be proposed. The advantage of this hypothesis is that it can encompass the findings of existing research. The specific binding of CGN to TLR does not depend on a single disaccharide unit; rather, the composite epitope is jointly constituted by the glycosidic framework of the polysaccharide backbone, the 3,6-anhydro bridge, the sulfate substitution pattern, and the higher-order spatial conformation adopted by the high-molecular-weight polysaccharide. Structural differences between CGN subtypes determine their TLR receptor preference.

Regarding the role of sulfate groups, the existing evidence is mainly derived from competitive binding assays with synthetic sulfated disaccharides and analogical inference. The sulfate groups of CGN are structurally similar to the phosphate groups of LPS, allowing for electrostatic interaction with the TLR4 binding domain [85] and exerting a non-specific electrostatic anchoring effect; subsequently, the composite recognition epitope, formed by the polysaccharide backbone glycosidic skeleton and the 3,6-anhydro bridge structure, is recognized by TLR4, thereby inducing receptor dimerization and activating downstream signal transduction [81]. Based on in vitro competitive binding experiments by Naïtaleb et al. using synthetic sulfated disaccharides [86], sulfate groups can bind to the basic positively charged ring region at the entrance of the MD-2 hydrophobic pocket, which overlaps considerably with the LPS phosphate-binding site. Synthetic sulfated disaccharides can mimic the LPS phosphate structure and competitively bind to the same basic region of the TLR4/MD-2 complex, with 6-O-sulfation being critical for maintaining binding activity. It should be noted that the above evidence is primarily derived from in vitro experiments with synthetic sulfated disaccharides; because the co-crystal structure of intact CGN polysaccharide with MD-2 has not yet been determined, its binding mode remains a hypothetical model. This sulfate group-mediated receptor activation pattern has been validated in studies of other sulfated polysaccharides [87].

### 3.2. Dual Signaling Pathways Downstream of TLR4: MyD88 and TRIF Branches

In 2008, Bhattacharyya et al. [48] explored the inflammatory pathways downstream of TLR4 in an in vitro model. Their study used λ-CGN with an undegraded molecular weight of approximately 1 × 10^6^ Da at a concentration of 1 μg/mL, and NCM460 cells, human primary colon cells, and mouse macrophages were used as experimental cell models. With this experimental system, they demonstrated for the first time that λ-CGN can target and bind to TLR4 on the surface of colonic epithelial cells and macrophages, activating the canonical TLR4 → MyD88 → IRAK → TRAF6 → MAPK/NF-κB inflammatory pathway [48]. Further validation using TLR4-knockout mice and wild-type mice as ex vivo models and λ-CGN treatment of isolated colonic tissues revealed that TLR4 deficiency significantly inhibited the secretion of the inflammatory factor KC, offering genetic evidence supporting this mechanism [48].

In 2020, Lopes et al. characterized the inflammatory pathway of low-molecular-weight κ-CGN (BDH product, catalog No.: 38,100; molecular weight not explicitly reported, in vitro concentration of 300 μg/mL) using in vitro and in vivo models. The in vitro experiments involved primary peritoneal macrophages isolated from Trif^−^/^−^ and Myd88^−^/^−^ knockout mice. Under these experimental conditions, κ-CGN promoted TNF and IL-1β production through the TLR4 pathway, and IL-1β maturation relied on the NLRP3/Panx1/P2X7 axis. For the in vivo studies, C57BL/6 mice were administered 5% low-molecular-weight κ-CGN by daily gavage for 20 consecutive days to establish a colitis model; the dose approximated 50–100 mg/kg per day, far exceeding that of typical human dietary exposure. This work identified a non-canonical TLR4 → TRIF → SYK → ROS → NLRP3 inflammatory pathway [53]. This MyD88-independent pathway recruits TRIF downstream of TLR4, induces ROS production via SYK phosphorylation, activates NF-κB and AP-1, and ultimately mediates IL-1β maturation and release via the NLRP3 inflammasome [53]. In their discussion, Lopes et al. analyzed the experimental data from McKim’s HEK293 recombinant reporter cell assay [83] and proposed that HEK293 cells lack a macrophage-specific membrane microenvironment and intracellular transport system, which makes it possible to produce false-negative results. Therefore, the findings cannot fully recapitulate the responses observed in primary immune cells. It is evident that when comparing and interpreting results from existing experiments on CGN biological activity, the inherent limitations and model-dependent features of each experimental system must be fully considered.

It must be emphasized that the conditions under which κ-CGN activates the downstream dual-branch pathway via TLR4, as revealed by Lopes et al., require careful qualification. First, the TRIF branch was only confirmed in mouse primary peritoneal macrophages [53], and no TRIF effect was observed in human colonic epithelial cell systems [48]. Second, the TRIF signaling reported by Lopes et al. only drove IL-1β gene transcription without activating IRF3 phosphorylation or inducing IFN-β production [53]. This differs from the classical LPS TRAM–TRIF–IRF3/type I interferon pathway. Therefore, the TLR4 signaling induced by κ-CGN represents a ligand-biased TLR4 agonistic effect and cannot be equated with the classical LPS TLR4 activation mode. Third, from the publication of this study to the search cutoff date of this review (30 April 2026), no independent original experiment has fully replicated the MyD88/TRIF dual-branch model of κ-CGN, nor has any original study directly negated this phenomenon; subsequent related studies have mostly cited this conclusion only [88,89] or merely validated the TLR4/NF-κB pro-inflammatory pathway [33] without performing Trif/Myd88 knockout, IRF3, or IFN-β detection, thus failing to independently verify the TRIF branch. Fourth, although recent studies in intestinal organoids from Crohn’s disease patients confirmed that κ-CGN upregulates IL-8 and TNF via TLR4, no MyD88/TRIF pathway splitting experiments were conducted [50], making it impossible to evaluate the contribution of TRIF. Therefore, the MyD88/TRIF dual-branch signaling pathway downstream of TLR4 should be described as a mechanism “confirmed under specific experimental conditions and awaiting independent replication.”

**Figure 2:**
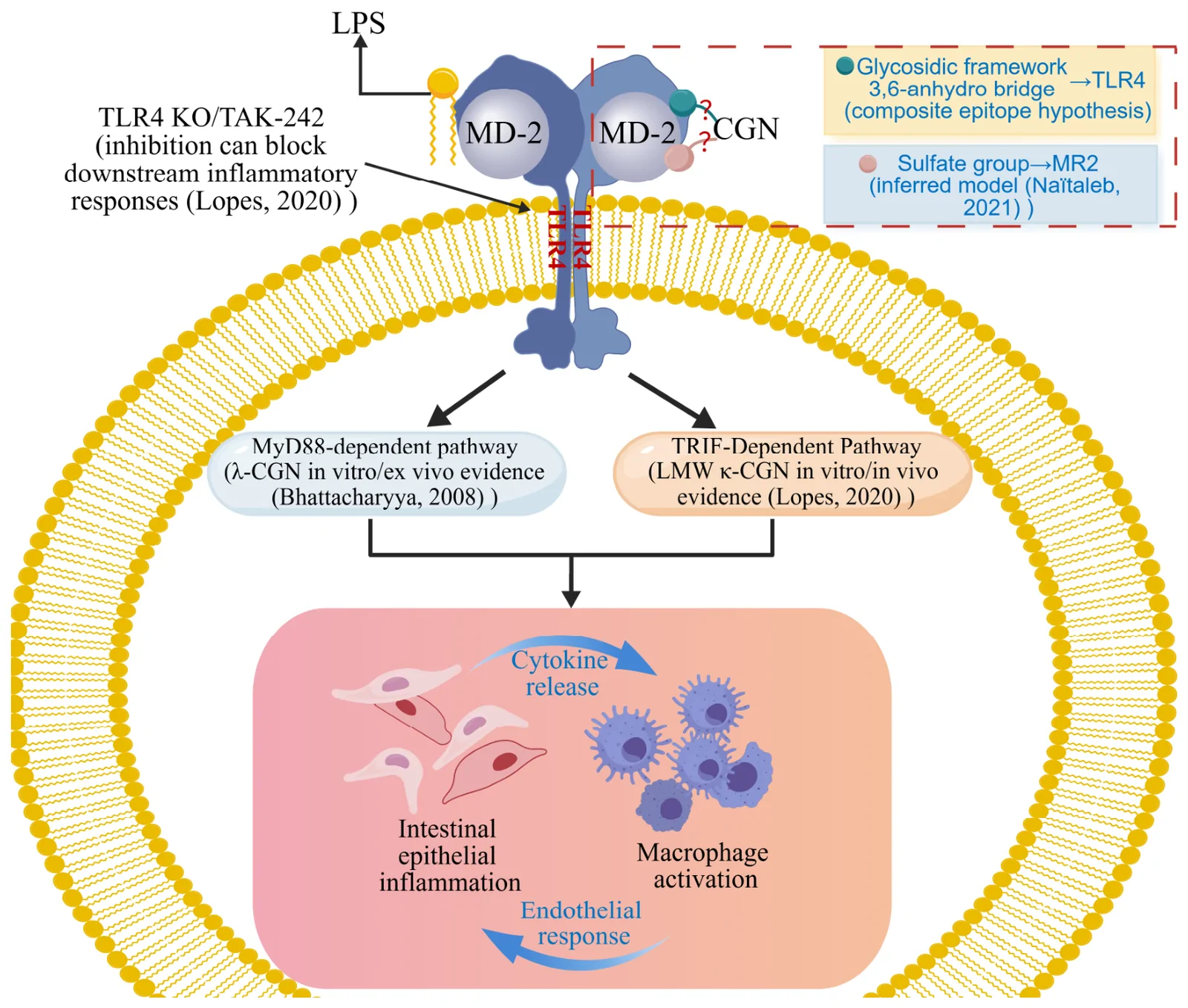
Schematic diagram of the hypothesized mechanism of TLR4-centered immune inflammatory activation. The sulfate groups of CGN first anchor to MD-2 [86]. The composite epitope formed by the glycosidic framework of the polysaccharide backbone and the 3,6-anhydro-bridge structure is then recognized by TLR4. This induces dimerization of TLR4, activating two downstream signaling pathways: the MyD88-dependent canonical pathway [48] and the TRIF-dependent non-canonical pathway [53]. These two pathways jointly promote intestinal epithelial inflammation, macrophage activation, and vascular endothelial responses. Downstream signals can be inhibited by TLR4 gene knockout or TAK-242 [53], indicating that TLR4 is the core upstream receptor in this process. LPS is shown as a reference in the figure. Red question marks and red dashed lines in the figure indicate that the binding between CGN and the TLR4 dimer remains unconfirmed and is speculative. Note: No co-crystal structure of the CGN and TLR4/MD-2 complex has been confirmed; the binding sites of CGN and TLR4/MD-2 remain a deduced model; the TRIF branch has only been confirmed in primary peritoneal macrophages from mice, with no replication in the literature.

The immune inflammatory activation mechanism centered on Toll-like receptor 4 is illustrated in Figure 2.

### 3.3. Involvement of TLR2/6 and Subtype Differences

In addition to TLR4, TLR2/6 also participates in the immune recognition of certain CGN subtypes, although the evidence is more limited and model-dependent compared with that of TLR4. Myers et al. (2019) [54] found in a HEK-293 cell transfection reporter system that λ-CGN primarily activates TLR2/6 and TLR4/6 heterodimers and does not activate the canonical TLR4 homodimer; its induced NF-κB activation mainly relies on TLR2/6 heterodimerization, with a secondary dependence on TLR4/6 heterodimerization. Silencing TLR6 significantly attenuates λ-CGN-induced inflammatory signaling, indicating that TLR6 is a key co-receptor.

At the in vivo level, Kim et al. observed in a rabbit model of hypercholesterolemia combined with subcutaneous injection of λ-CGN that the mRNA expression of TLR2 and TLR4 in aortic tissue increased in a gradient manner with the aggravation of inflammation, and the expression levels of both were closely correlated with the severity of atherosclerotic lesions, suggesting that the upregulation of TLR2 may reflect a secondary induction effect of TLR2 following TLR4 signaling activation under inflammatory conditions [90]. Although the κ-subtype has been reported to upregulate TLR2 mRNA expression in a fish model [91], this conclusion was derived from non-mammalian studies and did not involve TLR6. The existing evidence for TLR2/6 involvement comes primarily from the λ-subtype. Current research indicates that the receptor preference of different subtypes is related to differences in the primary and secondary conformations of CGN molecules, and their effects on inflammatory pathways still require systematic analysis.

According to the three-tier evidence stratification framework established in this paper, the experimental evidence presented in this section falls into Category C. These findings support mechanistic hypotheses and cannot be directly extrapolated to the safety assessment of food-grade CGN under routine human dietary exposure.

## 4. Mechanism of Inflammation-Mediated Vascular Endothelial Injury

In a mouse model receiving intraperitoneal κ-CGN injection (50 mg/kg) [29], CGN targeted TLR4 on the surface of peritoneal macrophages, initiating inflammatory signals and leading to the massive release of pro-inflammatory effectors such as TNF-α and IL-1β into the circulation. These mediators act on vascular endothelial cells, inducing a state of endothelial inflammatory pre-sensitization. A review by Baintner K. reported that peritoneal macrophages are key cells mediating CGN-induced inflammatory injury; specific depletion of peritoneal macrophages can significantly inhibit CGN-induced inflammatory ascites formation [92]. In a λ-CGN intraperitoneal injection mouse model (500 μg/mouse), the number of peritoneal leukocytes and neutrophils significantly increased, along with upregulation of IL-1β, IL-6, and oxidative stress markers, driving macrophage activation and neutrophil recruitment [27,28]. Activated macrophages release large amounts of proinflammatory mediators, chemokines, and nitric oxide, continuously activating the NLRP3/IL-1β inflammatory pathway while upregulating tissue factor (TF) expression, establishing a systemic inflammatory microenvironment that lays the pathological foundation for vascular endothelial injury and subsequent coagulation disturbances [28].

In Lee et al.’s mouse model, 24 h after the intraperitoneal injection of κ-CGN (1% solution, 50 mg/kg, Sigma, molecular weight not specified), TNF-α, IL-1β, and IL-6 were significantly elevated, eNOS phosphorylation levels decreased, and adhesion molecules such as ICAM-1, VCAM-1, P-selectin, and E-selectin, as well as thrombus-related factors including TXB2 and ET-1, were significantly upregulated, leading to thrombotic occlusion in the mouse tail vessels [29]. This study suggests that intraperitoneal injection of κ-CGN can induce systemic coagulation activation and consumptive coagulopathy. Hua Ji’an et al. [30] further verified that after Sprague Dawley rats received intraperitoneal injections of 50 mg/kg CGN for three consecutive days, prothrombin time (PT), activated partial thromboplastin time (APTT), and thrombin time (TT) were significantly shortened, while fibrinogen (FIB) levels were markedly increased, indicating that intraperitoneal injection of CGN can activate the coagulation system and induce consumptive blood coagulation disorders.

In the peritonitis model by Barth et al., the intraperitoneal injection of 1% CGN solution (250 μL, derived from *Chondrus crispus*, subtype unspecified) in BALB/c mice induced neutrophil recruitment, release of neutrophil extracellular traps (NETs), and robust ROS production, directly damaging the vascular endothelium and basement membrane; this release of NETs was independent of NADPH oxidase-derived ROS [31]. Activated neutrophils release toxic mediators such as elastase and cathepsin G, further exacerbating tissue damage [32,93,94]. A 2023 human clinical study [95] found that in AAV patients (systemic inflammatory state), neutrophil-derived MPO^+^ extracellular vesicles (MPO^+^ EVs) carry TF and H3Cit and are associated with enhanced thrombin generation, suggesting that under severe inflammatory stress, neutrophils can also participate in coagulation initiation by upregulating TF [95]. This provides clinical evidence for the mechanistic cascade of “neutrophil activation → NETs → tissue factor → coagulation initiation”. Nevertheless, the referenced study was not a CGN exposure study, and its outcomes and cannot be directly equated to the effects of CGN.

A large number of inflammatory factors can upregulate adhesion molecules such as VCAM-1, ICAM-1, and P-selectin on vascular endothelial cells, enhancing the adhesion of leukocytes to the vascular endothelium while promoting platelet adhesion and aggregation on the vessel wall, thereby accelerating thrombosis [29,96]. In a paw injection model, ROS-driven oxidative stress is an important pathway through which CGN disrupts the vascular endothelial barrier. In a λ-CGN-induced inflammatory pain model, a single injection of 100 μL of 3% λ-CGN (3 mg/paw, Sigma, molecular weight not specified) into the paw of female SD rats induced upregulation of oxidative stress markers in brain microvessels, disrupted the oligomeric assembly structure of the tight junction protein occludin, and increased vascular permeability; the ROS scavenger Tempol alleviated these injuries [21]. λ-CGN also induced an ROS burst in human umbilical vein endothelial cells, initiating a mitochondria-mediated apoptotic cascade [97]. Cross-species studies showed that after the subcutaneous injection of λ-CGN (0.5 mg/animal, single dose) into the footpad of cockatiels, footpad swelling did not return to baseline even after 84 days, suggesting that CGN-induced inflammation is not only an acute local reaction but may also lead to persistent tissue remodeling (such as fibrosis or scar formation) [26].

Inflammation not only directly impairs endothelial structure and function but also creates a systemic inflammatory milieu, triggering a distal coagulation cascade. Intraperitoneal injection of κ-CGN (100 mg/kg) induces multi-organ thrombosis in mice, accompanied by elevated TNF-α levels and activation of the HMGB1/NF-κB/NLRP3 pathway in lung tissue [33]; in another model, intraperitoneal injection of CGN (50 mg/kg) shortened APTT, PT, and TT while elevating FIB, confirming that systemic inflammation can trigger the coagulation cascade [34]. These experiments provide direct evidence for the “inflammation–coagulation” coupling induced by CGN.

**Figure 3:**
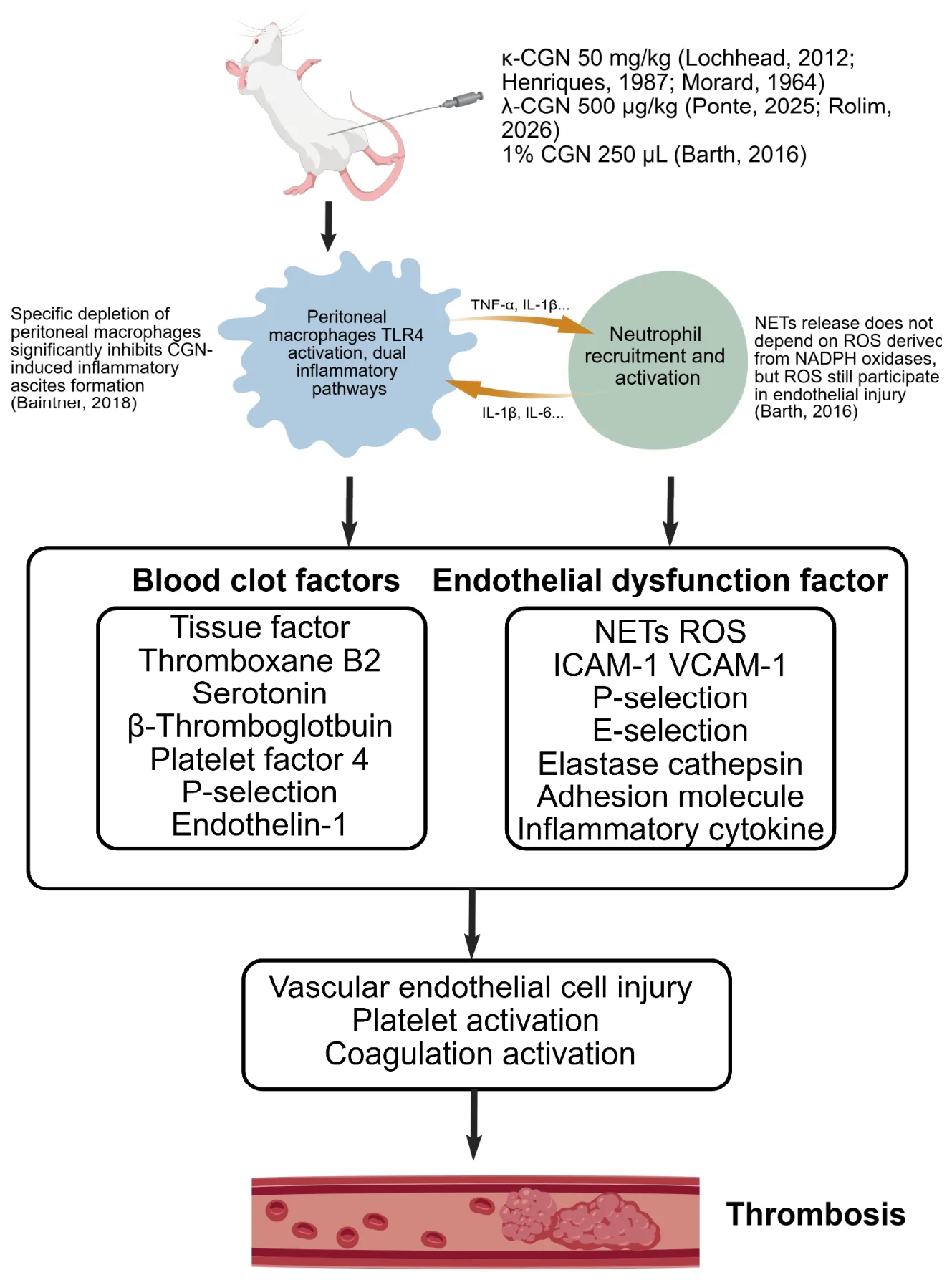
Schematic diagram of the intraperitoneal injection of carrageenan initiating the inflammation–endothelial injury–thrombosis pathway via TLR4. Intraperitoneal injection of CGN activates TLR4 on the surface of peritoneal macrophages [21,22,27,28,29,30,31,32,98], triggering a dual inflammatory cascade and the release of pro-inflammatory mediators, including TNF-α and IL-1β. These mediators recruit and activate neutrophils, which secrete effector molecules such as NETs, ROS, IL-1β, and IL-6. Notably, NET release is independent of NADPH oxidase-derived ROS [31]. Crosstalk between macrophages and neutrophils and other immune cells produces abundant coagulation factors and mediators of endothelial dysfunction, generating a systemic inflammatory milieu. This milieu damages the vascular endothelium, activates platelets, and initiates the coagulation cascade, ultimately resulting in thrombosis. Specific depletion of peritoneal macrophages markedly attenuates CGN-elicited inflammatory ascites [92], verifying that macrophages act as the core initiator within this pathway. This schematic represents a conceptual mechanistic framework integrated from heterogeneous experimental evidence. All supporting data originate from intraperitoneal administration studies and cannot be directly extrapolated to routine oral dietary exposure in humans.

Peritoneal macrophages serve as the key cellular player in CGN-induced inflammation–endothelial injury–thrombosis following intraperitoneal administration; this cascade mechanism is depicted in Figure 3.

CGN-triggered thrombosis following intraperitoneal administration is also linked to gut microbiota dysbiosis. After ICR mice were intraperitoneally administered 0.2% carrageenan (approximately 20 mg/kg/day) for 10 consecutive days, 16S rRNA sequencing revealed markedly reduced gut microbial diversity and an elevated Firmicutes/Bacteroidetes ratio. Serum concentrations of TNF-α, IL-6, NF-κB, and IL-1β were increased. Colon tissue showed downregulated mRNA expression of antioxidant enzymes (SOD and CAT) alongside upregulated pro-inflammatory cytokine transcripts. Expression of vascular adhesion molecules (ICAM-1, VCAM-1, and E-selectin) in the tail vein was increased, accompanied by leukocyte infiltration and platelet aggregation [37]. Similarly, intraperitoneal injection of κ-CGN (50 mg/kg) in Kunming mice induced gut microbiota dysbiosis (decreased Firmicutes; increased Muribaculaceae), concurrent with reduced plasma 1-PA and D-dimer levels and elevated vWF and TXB2 [36]. These findings indicate that systemic inflammation elicited by intraperitoneal CGN can worsen dysbiosis and amplify vascular endothelial injury and the procoagulant state via bidirectional “gut–vascular axis” crosstalk, forming a vicious cycle of “inflammation–dysbiosis–endothelial injury–thrombosis”. Probiotic intervention with *Lactiplantibacillus plantarum* CQPC03 (10^9^ CFU/kg) can reverse dysbiosis, lower inflammatory markers, and shorten thrombus length [37].

The “inflammation–endothelial injury–coagulation activation–thrombosis” cascade outlined in this section is a conceptual synthesis of evidence from different experimental systems, different CGN subtypes and molecular weights, different doses, and different routes of administration (intraperitoneal injection, footpad injection, intravenous injection, etc.). It is not a linear pathway validated by a single experiment. This cascade may support mechanistic interpretation and hypothesis generation. The strength of the evidence for each step varies; some was obtained from gene knockout mice and in vivo models, some from in vitro cellular assays, and some from cross-species observations. Based on the three-tier evidence stratification framework established in this paper, the experimental evidence presented in this section falls into Category B/Category C. Accordingly, it cannot be directly extrapolated to routine oral dietary exposure in humans.

## 5. Activation of the Intrinsic Coagulation System and Anatomical Basis of Tail Thrombosis

### 5.1. Activation of the Intrinsic Pathway of Coagulation

In 1964, Morard JC first reported in *Nature* the establishment of a complement activation-mediated renal inflammatory injury model established via tail vein injection of CGN [98]. In 1979, Steele further refined this modeling protocol, systematically characterized multi-organ pathological injury induced by carrageenan and its underlying molecular regulatory mechanisms [41], confirming that it is capable of triggering the intrinsic coagulation pathway by activating blood coagulation factor XII. That study used CBA inbred mice as the research subjects. After tail vein injection of 0.9 mg of λ-CGN, blood coagulation factor XII was activated, triggering the cascade activation of the coagulation system, kinin system, and complement system, leading to widespread microvascular embolism and platelet aggregation throughout the body, which ultimately resulted in ischemic necrosis of the liver and myocardium. Concurrently, the study revealed that carrageenan can secondarily induce macrophage phagocytosis and activation, as well as lymphocyte immune activation, mediating the sustained activation of blood coagulation factor XII (FXII) and prolonging the vascular injury process [41].

CGN-induced coagulation upon intravenous administration can occur independently of endothelial injury and tissue factor (TF) involvement. Based on cell-free biochemical experiments, the sulfate ester groups of CGN can electrostatically interact with positively charged amino acid residues on the surface of FXII, with binding affinity positively correlated with the degree of CGN sulfation [99]. Specific binding of CGN to FXII induces conformational rearrangement of FXII, driving its autoactivation into FXIIa. FXIIa, as the initiating factor of the intrinsic pathway of coagulation, sequentially activates downstream coagulation factors centered on coagulation factor XI [100]. The aforementioned evidence of a CGN–FXII interaction is primarily derived from cell-free biochemical binding experiments, and its occurrence in the plasma environment and physiological relevance require further in vivo studies for confirmation. This mechanism is only applicable when CGN directly enters the blood circulation (e.g., via intravenous injection). Based on the three-level evidence stratification framework established in this paper, the experimental evidence in this paragraph falls into Category C.

Yokomori R et al. employed a κ-carrageenan intraperitoneal injection tail thrombosis model using platelet-specific CLEC-2 conditional knockout mice, together with in vitro platelet assays with JAQ1 (GPVI-blocking antibody), and identified a GPVI-independent platelet prothrombotic pathway [35]. Surface plasmon resonance (SPR) assays from this study verified that κ-CGN directly binds to platelet CLEC-2 as an exogenous ligand, activating the downstream Src/Syk/PLCγ2 signaling axis and inducing platelet activation and a procoagulant phenotype [35]. Using CLEC-2 knockout mice in an intraperitoneal injection model, the authors demonstrated that κ-CGN (molecular weight unspecified) activates platelets through the platelet CLEC-2 receptor; CLEC-2 ablation abolishes this effect. Following intraperitoneal κ-CGN administration, the tail thrombosis rate was 69.27% in wild-type mice, whereas this value dropped to 15.59% in the knockout mice (*p* < 0.0001) [35]. This study also included a tail vein injection model and confirmed that CLEC 2 gene knockout reduces the rate of thrombosis in this mouse model [35]. All experiments adopted non-oral administration routes, and the physiological relevance of CLEC-2 signaling following oral exposure remains unclear. The authors also stated the following in their discussion: “Intact macromolecular CGN is hardly digested in the intestine and is unlikely to enter the human blood circulation under normal conditions.” Accordingly, CLEC-2-mediated platelet activation should be described as “a mechanism validated under intravenous or intraperitoneal injection conditions”. Based on the three-level evidence stratification framework established in this review, the experimental evidence for CLEC-2-mediated platelet activation presented in this paragraph belongs to Category C.

### 5.2. Anatomical Basis of Tail Vein Thrombosis

The CGN-induced rodent tail thrombosis model is a widely used in vivo model for antithrombotic drug screening and mechanistic research [37,101,102]. Its core feature is that after intraperitoneal or intravenous injection of CGN, thrombi are not uniformly distributed throughout the systemic vasculature but specifically form significant ischemia, dark discoloration, and necrosis at the distal end of the tail, a characteristic closely related to the unique vascular anatomy of the tail.

Wang et al. [103] systematically analyzed the fine structure of the mouse tail vasculature using ink perfusion, Micro-CT three-dimensional reconstruction, and histological sectioning. The results showed that the mouse tail vasculature originates from the distal sacroiliac branches of the abdominal aorta: the median caudal artery is a direct continuation of the median sacral artery distally, while the left and right lateral caudal arteries arise from the inferior gluteal artery branching off the common iliac artery. The tail possesses two interrelated circulatory architectures: the median and bilateral lateral vessels form the longitudinal main circulation, while the transverse vessels surrounding each caudal vertebra and deep microvessels constitute segmental local microcirculation. The two types of circulation are connected only by slender transverse anastomoses, with extremely limited compensatory capacity [103]. Wang Lei et al. [104] also confirmed the existence of the above three longitudinal systems, segmental transverse arteries, and superficial and deep communicating vessels in rat tails, indicating that the vascular structure of rodent tails is highly conserved.

The tail is located at the most distal end of the systemic circulation, with blood flowing unidirectionally from the tail base to the tip. The distal region has small vessel calibers and sparse anastomotic branches and is naturally in a state of low perfusion and low shear stress, making it prone to retaining procoagulant substances. Anatomically, the superficial subcutaneous longitudinal main veins lack valve structures, with only the deep dorsal tail vein containing a small number of functional venous valves [103,104]. Coupled with gravity and low flow velocity, blood stasis in the distal region is further exacerbated. The tail skin is thin, subcutaneous fat is minimal, and local temperature is low, all of which are physical factors conducive to maintaining thrombin activity. Local microcirculation is based on individual caudal vertebrae as independent functional units, and the slender anastomotic branches between segments cannot achieve effective cross-segment compensation. Once microvascular thrombosis occurs, the ischemic area tends to spread continuously toward the tail tip, presenting a characteristic purple-black necrotic phenotype. In contrast, internal organs such as the liver, lungs, and kidneys, due to high-pressure continuous blood flow, abundant valves, thick-walled vessels, and dense network shunts, can effectively resist CGN-induced procoagulant injury, showing only a small amount of focal microthrombi without extensive progressive thrombosis [29]. These anatomical features are unique to the mouse/rat tail thrombosis model and naturally differ from human vascular anatomy or the mechanisms of human thrombosis. The experimental results from the tail vein thrombosis model cannot be directly extrapolated to human thrombosis.

There are significant differences in the thrombotic initiation mechanisms between intraperitoneal injection and tail vein injection as two drug administration routes. After a tail vein injection, CGN directly enters the blood circulation, and its negatively charged sulfate groups bind to coagulation factor XII (FXII), activating the intrinsic pathway of coagulation (see Section 5.1); it can also directly activate the platelet CLEC-2 receptor [35]. The longitudinal distribution of tail vessels allows CGN to rapidly spread along the entire tail length, while the double-layer cage-like structure and abundant collaterals enable the coagulation cascade to initiate simultaneously at multiple segments. Additionally, the thin tail skin, low subcutaneous fat, and relatively low local temperature further promote coagulation activation. These anatomical features collectively explain why tail infarction can be observed within minutes after tail vein injection of CGN [38,39,40,80], with lesions showing distinct tail segment specificity.

In contrast, intraperitoneally injected CGN does not enter circulation directly but instead activates systemic inflammatory pathways via peritoneal macrophages (see Section 4). Circulating inflammatory cytokines extensively damage the endothelium throughout the body; only the tail, due to its anatomical characteristics of distal stasis, valve defects, thin-walled microvessels, and weak collaterals, becomes a specific target area for preferential thrombus colonization and progression. Therefore, tail thrombosis after intraperitoneal injection is delayed by approximately 3 h compared with thrombosis following tail vein injection, gradually appearing 3 h after administration and reaching a significant level at 24 h [29,33,34,36], occurring to a milder degree.

In summary, tail vein injection rapidly induces thrombosis by directly activating coagulation factors and platelet receptors, while intraperitoneal injection remotely damages the endothelium via inflammation cytokines, leading to secondary thrombosis at anatomically vulnerable sites. The differences in dynamics and primary targets between the two routes stem from the unique vascular anatomy of the tail.

## 6. In Vivo Process of Oral Carrageenan and Colitis Mediated by the Gut–Blood Axis

### 6.1. Gut Microbiota-Mediated Degradation, Barrier Disruption, and NLRP3 Activation

It should be emphasized that the cascade described below is observed under defined experimental model conditions and does not represent proven outcomes for conventional human dietary CGN intake. When the intestinal epithelial barrier is intact, food-grade CGN (≥100 kDa) cannot readily cross the intestinal epithelium into the circulation in substantial quantities due to its large molecular size and strong negative charge; intestinal digestive enzymes also cannot effectively degrade CGN [105,106]. Bacteroides species in the gut that harbor carrageenase (e.g., *Bacteroides xylanisolvens*) can secrete β-carrageenase and sulfatase, gradually cleaving high-molecular-weight CGN into low-molecular-weight degradation fragments (dCGNs) [52]. When intestinal epithelial barrier permeability increases, dCGNs can reach macrophages in the lamina propria of the submucosa, where they are endocytosed and accumulate in lysosomes, inducing lysosomal dysfunction; lysosomal membrane permeabilization provides the second signal required for NLRP3 inflammasome activation [52,65,107]. This oral intestinal model exhibits a clear temporal response sequence: intestinal epithelial cells first sense luminal CGN and continuously secrete chemokines such as IL-8 via the TLR4–Bcl10 pathway, recruiting monocytes to infiltrate the lamina propria; only after macrophages encounter dCGN is the two-stage NLRP3 signaling cascade fully initiated.

A literature analysis reveals that our current understanding of the phenomenon of “gut microbiota degrading food-grade CGN into low-molecular-weight fragments and subsequently inducing inflammation” is based primarily on in vitro gut fermentation, animal models, and cell experiments [52,65,107]; there is still a lack of quantitative in vivo human tracing studies demonstrating that “under conventional dietary exposure, 20–40 kDa dCGN can be generated in the gut and reach biologically meaningful concentrations.” Accordingly, this process should be described as a mechanism that “can occur under experimental conditions but remains to be verified under conventional human dietary exposure,” rather than an established consequence of human exposure.

However, subsequent studies have shown that low-molecular-weight fragments can be produced under the action of gut microbiota [52,108,109], and trace absorption (approximately 5–10%) was observed in high-dose animal experiments [43,105]. Therefore, the above “difficult to penetrate” conclusion should be limited to conditions of intact epithelium and normal dietary exposure.

Regarding the effects of oral CGN on the intestinal barrier, different in vitro models have yielded inconsistent results. In Caco-2/HT29-MTX co-cultures (containing a mucus layer; without immune cells), treatment with κ-, ι-, and λ-CGN (food-grade, 100 μg/mL) for 24 h did not alter transepithelial electrical resistance (TEER), suggesting that CGN does not directly disrupt the epithelial barrier [50]. However, in Caco-2/THP-1 co-cultures (containing macrophage-like cells; without a mucus layer), treatment with κ-CGN (1–10 μg/mL) for 4–24 h induced a sustained reduction in TEER and a significant increase in Caco-2 cell apoptosis (late apoptosis 12.4% → 38.2%), accompanied by substantial increases in TNF-α, IL-1β, and IL-6. Blockade using an anti-TNFR1 antibody reduced apoptosis by approximately 57%, confirming TNF-α as the core mediator [51]. The discrepancy between the two studies’ outcomes may be due to model differences: the mucus layer derived from HT29-MTX can weaken direct contact between CGN and the epithelium, while macrophages derived from THP-1 amplify TNF-α-dependent inflammatory damage. Additionally, in colonic organoid-derived monolayers from Crohn’s disease (CD) patients, κ-CGN (100 μg/mL) also did not alter TEER but upregulated TJP1/ZO-1 and OCLN expression (suggesting compensatory barrier reinforcement), with more pronounced pro-inflammatory effects under inflammatory conditions [50]. These in vitro findings provide mechanistic clues that oral CGN may exacerbate inflammation in the context of an impaired intestinal barrier or inflammatory microenvironment, but due to the large disparity between exposure concentrations and simplified systems compared with actual dietary scenarios, they cannot be directly extrapolated to dietary safety assessments for the general population. Based on the three-level evidence stratification framework established in this review, the above-mentioned experimental evidence falls into Category C.

### 6.2. Inflammatory Amplification and Systemic Effects

Following physical disruption of the intestinal barrier, CGN crosses the mucus layer and activates the TLR4/NF-κB and NLRP3 pathways in lamina propria macrophages and dendritic cells. This triggers the release of pro-inflammatory factors including TNF-α, IL-1β and IL-6, creating a vicious cycle of “barrier damage–endotoxin leakage–TLR4 overactivation–exacerbated inflammation” [16,17]. In a TNBS-induced colitis model using BALB/c mice, 14-day pretreatment with κ CGN increased weight loss and mortality, aggravated colonic inflammation, decreased the proportion of regulatory T cells and IL-10 levels, and upregulated the expression of IL-2, TNF-α, IL-6, TLR4, NF-κB, p-ERK, p-JNK, p-Jun, IL-8, and MDA [42]. However, the dose was not specified in this study, and these findings cannot be directly extrapolated to normal dietary exposure.

Fath et al. provided CF1 mice with drinking water containing 10% degraded CGN (d CGN, non-food-grade) for 10 days and successfully induced bloody diarrhea, pericryptal inflammation, colonic mucosal erosion with hemorrhage, and precancerous lesions [110]. This model employed a high concentration of non-food-grade degraded CGN; therefore, its conclusions cannot be extrapolated to food-grade CGN. In a zebrafish enteritis model, feeding a diet supplemented with 0.1% κ CGN for 10 consecutive days induced intestinal luminal dilation, mucosal thinning, irregular villous morphology, increased goblet cell numbers, and leukocyte infiltration. These changes were accompanied by elevated TNF-α, COX-2, IL-1β, and IL-8 levels, alongside gut microbiota dysbiosis [45]. These observations originate from non-mammalian models and high concentration exposure; they similarly cannot directly reflect the safety of this food additive for humans.

### 6.3. Pharmacokinetics, Toxicity Characteristics, and Limitations of Safety Conclusions

Metabolic data describing systemic exposure following oral CGN administration have primarily been obtained for low-molecular-weight fragments (<50 kDa) [108,109]. After oral administration of acid hydrolyzed κ CGN (non-food-grade degraded CGN) to mice, peak plasma concentrations were detected, excretion occurred via urine, and tissue accumulation was observed in the liver and kidneys; the overall absorption rate was estimated to be 5–10% [43,105]. By contrast, no measurable systemic absorption of intact food-grade CGN (≥100 kDa) was observed in that same study. Studies using FITC-labeled acid-hydrolyzed κ CGN reported peak plasma concentrations at 3 h post oral administration, a mean residence time of 36.6 h, 9.2% urinary excretion, and distribution within hepatic and renal tissues. After 14 days of repeated dosing, hepatic necrosis, renal tubular vacuolation and incomplete colonic epithelial integrity were noted [43]. Nevertheless, FITC labeling may alter the physicochemical properties of CGN, and the test substance was not food-grade CGN. Therefore, its pharmacokinetic parameters cannot directly reflect the behavior of food-grade CGN under routine dietary exposure.

The conclusion that food-grade CGN is “poorly absorbed and exhibits no substantial toxicity under normal dietary exposure” is primarily supported by animal experiments performed at relevant dietary exposure levels (typically <50 mg/kg/d) under conditions of an intact intestinal barrier [60,66,67,68]. This represents the consensus within the research community and food safety regulatory bodies. On the other hand, datasets supporting the claim that “CGN exerts toxic effects” generally originate from four categories of experimental settings: (1) high oral CGN doses (>100 mg/kg/d or >5% feed inclusion) [16,17,48], (2) in vitro cell assays using high concentrations (100–300 μg/mL) [48,49,51,53], (3) low-molecular-weight degradation fragments (poligeenan or LMW-CGN, <40 kDa) [52,65], and (4) non-mammalian models such as zebrafish [45]. These observations reveal potential proinflammatory mechanisms of CGN under defined experimental conditions. Based on the three-level evidence stratification framework established in this paper, these experimental data are categorized into Category B. Such findings are limited to mechanistic exploration and should not be directly extrapolated to safety assessments for human routine dietary exposure nor directly adopted as evidence for food additive risk assessment.

Based on the available data and defined usage conditions, the EFSA, FDA, and JECFA regard food-grade CGN as safe under normal dietary circumstances and have set a temporary acceptable daily intake or concluded that specifying an ADI is unnecessary [60]. This regulatory conclusion should be interpreted as “under restricted usage conditions and with currently available evidence, consistent severe adverse effects have not been identified” rather than as the “absolute exclusion of all potential inflammatory or thrombotic effects across all experimental and physiological scenarios”.

Available evidence supports that food-grade CGN does not trigger marked proinflammatory responses or prothrombotic risks under conventional dietary exposure. However, several uncertainties persist: direct human tracer data are lacking regarding the yield, the molecular weight distribution and exposure concentration of low-molecular-weight fragments generated via gut microbiota-mediated degradation are unknown, interindividual susceptibility differences (e.g., patients with inflammatory bowel disease, irritable bowel syndrome or impaired intestinal barrier function) remain unvalidated by large clinical cohorts, and variations in experimental exposure conditions (dose, administration route, disease subtypes, molecular weight, and model system) can substantially modify study conclusions.

A comparison of the in vivo processes and toxicity characteristics of different types of carrageenan is provided in Table 3.

**Figure 4:**
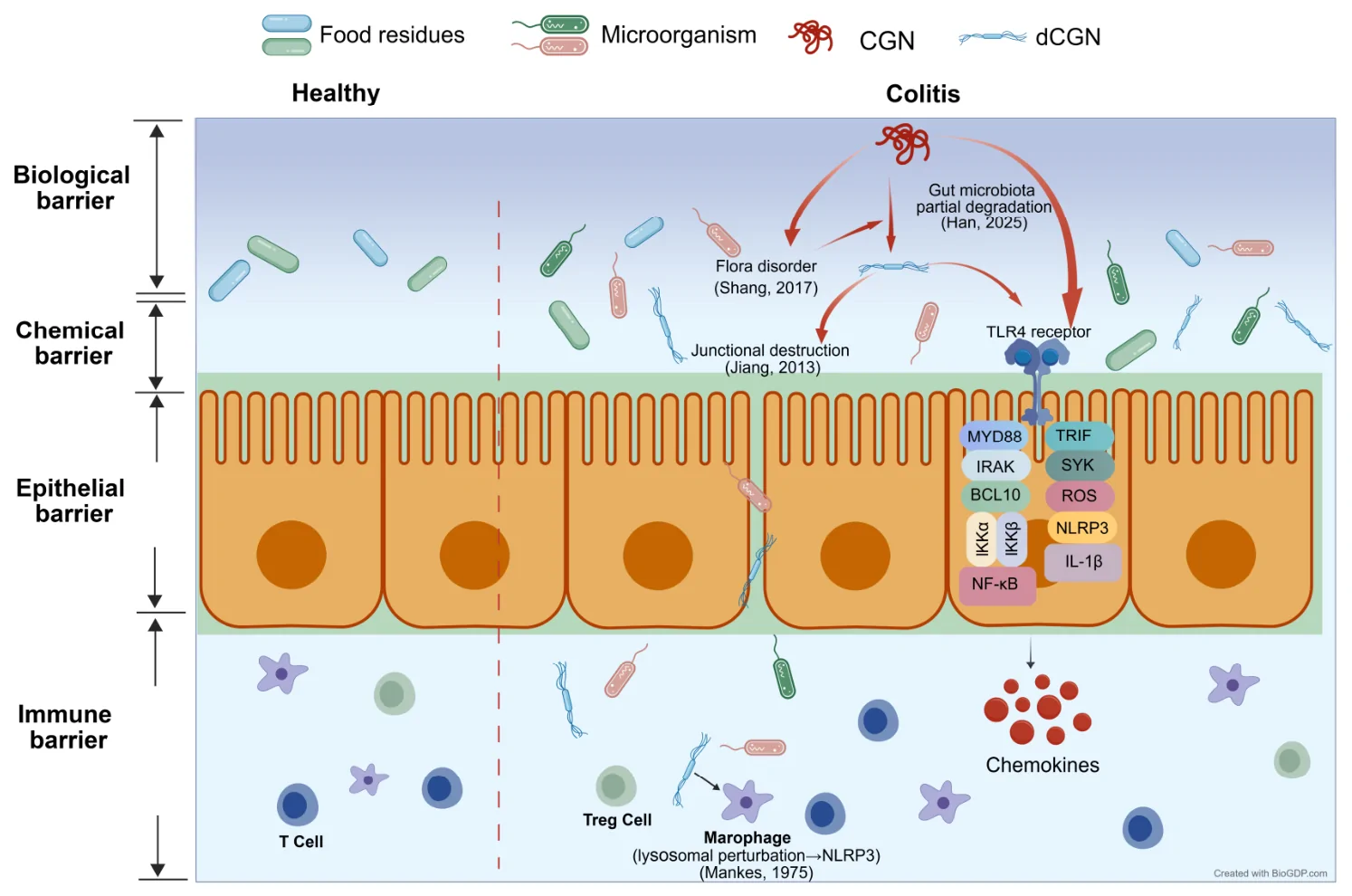
Schematic diagram of the proposed mechanism of oral carrageenan-induced colitis. After CGN enters the gut, the gut microbiota degrades CGN into low-molecular-weight pro-inflammatory fragments [52] and concurrently triggers gut dysbiosis [49,111]. CGN also increases oxidative stress within intestinal tissues, disrupting tight junctions between intestinal epithelial cells [51]. Once intestinal barrier function is impaired, intact CGN and/or dCGN can reach immune cells in the lamina propria, activating TLR4-receptor-mediated dual inflammatory pathways [48,53], stimulating pro-inflammatory cytokine release and potentially driving intestinal inflammation. Macrophage uptake of dCGN and subsequent lysosomal perturbation participate in NLRP3 inflammasome activation [107]. This figure represents a conceptual synthesis built upon heterogeneous evidence derived from in vitro, in vivo animal, and human ex vivo studies and should not be interpreted as a fully validated pathway operative in humans. The relative contributions of the MyD88 and TRIF signaling branches remain unclear; the TRIF branch has only been verified in mouse peritoneal macrophages [53].

Figure 4 illustrates the proposed mechanism through which carrageenan (CGN) induces colitis following oral ingestion into the intestinal tract.

### 6.4. Mechanistic Differences Between Oral Intestinal Model and Intraperitoneal Injection Model

The oral intestinal model and intraperitoneal injection model share a common feature: CGN stimulates macrophages through TLR4 to provide the priming signal for NLRP3, ultimately promoting the secretion of mature IL-1β. Nevertheless, the effective stimulatory molecules, barrier status, sources of intracellular second signals and the onset kinetics of inflammation differ substantially. Hence, experimental conclusions cannot be directly translated between these two models.

In the oral intestinal model, high-molecular-weight CGN is progressively degraded in situ by gastrointestinal microbiota to produce dCGN. When the mucosal barrier is impaired, dCGN reaches lamina propria macrophages beneath the epithelium and contributes the second signal for NLRP3 activation via lysosomal perturbation. The stimulus comprises a mixture of “high-molecular-weight parent CGN + continuously in situ generated dCGN”. Inflammatory signaling remains localized within the submucosa, manifesting as chronic, low-grade local inflammation [52,65,107].

In the intraperitoneal injection model, there is no epithelial barrier blockade nor preprocessing via microbial degradation. High-molecular-weight CGN directly contacts TLR4 expressed on the surface of peritoneal macrophages, and macrophage activation marks the onset of inflammation. In this system, NLRP3 activation depends more heavily on the TLR4–TRIF–Syk pathway, intracellular ROS, and P2X7 mediated potassium efflux. High-molecular-weight CGN is not readily endocytosed efficiently into lysosomes; accordingly, the contribution of the lysosomal perturbation pathway is relatively limited [53]. Intraperitoneally administered CGN rapidly recruits abundant neutrophils [66,93] and triggers NETosis [31], creating an acute inflammatory microenvironment. This phenotype is fundamentally distinct from the chronic low-grade inflammation of the intestinal mucosa induced by oral CGN, and findings regarding NETosis cannot be directly extrapolated to the oral intestinal setting.

Therefore, when assessing the intestinal safety of food-grade CGN, priority should be assigned to the oral intestinal model, which incorporates the combined effects of microbiota mediated degradation and changes in epithelial barrier permeability.

## 7. Carrageenan-Induced Inflammation–Thrombus Animal Model

### 7.1. Local Acute Inflammation Model of Paw Edema

In 1962, Winter et al. first established the acute paw edema model in rats by injecting carrageenan (CGN) into the hind paw [13], which later became the gold standard for primary screening of anti-inflammatory drugs. In 1987, Henriques et al. demonstrated that this model induces biphasic swelling and developed a standardized mouse CGN inflammation model [22]. In 1999, Nantel et al. further elucidated that the model sustains COX-2/PGE_2_-positive feedback regulation [23]; in 2004, Posadas et al. identified strain-dependent differences in biphasic profiles and inflammatory mechanisms across mouse strains [24]. Studies using this model have shown that Melastomataceae extract downregulates TNF-α and IL-1β via the inhibition of NF-κB nuclear translocation [112]; astragalin suppresses the NF-κB pathway, reduces iNOS, COX-2, and MCP-1 expression, enhances antioxidant enzyme activity, and ameliorates oxidative stress and histopathological injury in paw tissue [25].

This model mainly reflects local acute inflammation and oxidative stress. Its underlying mechanisms partially overlap with the upregulation of ROS, NF-κB, and adhesion molecules described in Section 4, but thrombosis is not involved.

### 7.2. Local Thrombosis Model

In 1973, Giroud et al. first reported CGN-induced thrombosis [14]; in 1985, Bekemeier et al. established a mouse tail thrombosis model (“black tail model”) via intraperitoneal κ CGN injection [80], which has since become a classic model for investigating local inflammation-associated thrombosis. This model exhibits a clear phenotypic readout: purplish dark tail swelling, segmental black discoloration, and tissue necrosis, enabling visual observation and convenient quantitative analysis. Using this model, researchers have confirmed that Antarctic krill oil interrupts the “endothelial injury–inflammatory activation–platelet aggregation–coagulation activation–thrombosis” cascade by inhibiting platelet activation, protecting the vascular endothelium and suppressing local inflammation [29]. Gegen Qinlian Pills reduce TNF-α and IL-1β secretion by downregulating HMGB1 and blocking the NF κB/NLRP3 pathway [33].

This model directly supports the “inflammation–endothelial injury–coagulation activation–thrombus” coupling framework described in this paper. However, thrombus colonization in this model is highly dependent on the special vascular anatomy of the rodent tail (distal hypoperfusion, valve defects, and weak collateral compensation) and cannot be equated with human thrombotic diseases.

### 7.3. Chronic Gastrointestinal Inflammation and Colitis Models

Through their research from 1969 to 1971, Watt and Marcus first demonstrated that the oral administration of degraded CGN can induce colonic ulceration in guinea pigs [62,63]. In 1984, Fath et al. [44] further confirmed that degraded λ CGN serves as the optimal inducer for model establishment. Administration of 10% degraded λ CGN to CF1 mice for 10 consecutive days induces bloody diarrhea, pericryptal inflammation and colonic mucosal erosion with hemorrhage. Histological features include crypt architectural distortion, lamina propria inflammatory infiltration and ulcer formation, which are most prominent in the proximal colon. Concurrent findings include aberrant colonic epithelial proliferation and precancerous lesions (proliferative zone extending to the upper one third of the intestinal glands) [44]. The pathological features of this model closely resemble human ulcerative colitis, rendering it a widely used tool for exploring intestinal inflammatory mechanisms and screening anti-inflammatory agents. Research based on this model shows that *Artemisia alba* extract (500 mg/kg) markedly alleviates colitic injury, preserves colon length and repairs mucosal architecture [110]; chestnut shell extract downregulates TNF-α, COX-2, IL-1β, and IL-8 while upregulating IL-10 and modulates gut microbiota composition [45].

This model supports the mechanism whereby oral CGN administration triggers intestinal inflammation via microbial degradation, barrier disruption, and TLR4/NLRP3 activation and serves as an important experimental system for assessing the intestinal safety of food-grade CGN.

### 7.4. Acute Muscle Inflammation Model

An acute muscle inflammation model can be established by injecting λ CGN (0.1 mL, 3 mg/mL) into the rat gastrocnemius muscle [46]. Using this model, investigators found that *Chaenomeles speciosa* extracts (5.25, 10.5 mg/mL), with oleanolic acid and ursolic acid as the main active constituents, promote muscle fiber repair by scavenging ROS and inhibiting the NF-κB pathway. These extracts downregulate TNF-α, IL-1β, and IL-6, upregulate TGF-β, reduce serum lactate dehydrogenase and creatine kinase levels, mitigate muscle fiber tearing and local inflammatory cell infiltration, and effectively improve limb muscle strength in rats [46].

This model does not directly involve thrombus formation, yet it demonstrates the common ability of CGN to trigger inflammation, oxidative stress, and NF-κB pathway activation in local tissues, consistent with the ROS/NF-κB component of the endothelial injury mechanism described in Section 4. It provides a drug-screening dimension for the local inflammation tool model.

### 7.5. Subcutaneous Air Pouch Inflammation Model

After injecting sterile air into the dorsal subcutaneous tissue of mice to create an air pouch, acute local inflammation can be induced on day 7 by injecting 0.1 mL of 1% λ CGN solution (approximately 1 mg/kg) into the pouch. This model permits simultaneous evaluation of inflammatory progression and resolution mechanisms [47]. Studies using this model demonstrate that methanol extract of *Cariniana rubra* stem bark exerts anti-inflammatory effects by inhibiting neutrophil and monocyte recruitment, lowering TNF-α expression, and upregulating the endogenous anti-inflammatory protein Annexin A1 (AnxA1) [47].

This model involves leukocyte recruitment, TNF-α release, and mechanisms governing inflammation resolution. Impaired inflammation resolution is an important factor contributing to the prethrombotic state, thus serving as an indirect supplement to the inflammation–thrombosis coupling framework.

### 7.6. Overall Positioning and Translational Limitations of the Models

Based on the three-level evidence-stratification framework established in this paper, all models outlined above fall into Category B and serve as experimental tool models. Findings from these models are restricted to mechanistic exploration and efficacy screening and cannot be directly extrapolated to safety assessments of food-grade CGN under routine human dietary exposure.

## 8. Conclusions

This review applies an author-defined conceptual three-tier evidence-stratification framework (not a formally validated standardized evidence-grading system) to integrate evidence concerning carrageenan (CGN)-triggered immune inflammation and thrombosis according to molecular weight, subtypes, administration routes, and evidence hierarchy. It facilitates clear differentiation between safety evidence for food-grade CGN under routine dietary exposure and mechanistic findings obtained under specific experimental conditions and prevents direct extrapolation of conclusions derived from the latter to the safety assessment of food additives.

Existing evidence supports that food-grade high-molecular-weight CGN (≥100 kDa) cannot penetrate the intestinal epithelium in large quantities when the intestinal barrier remains intact under routine dietary exposure. Under defined usage conditions, regulatory evaluations and animal studies have not identified marked pro-inflammatory or pro-thrombotic risks associated with food-grade CGN. Food-grade CGN, LMW-CGN/d-CGN, and PGN differ fundamentally in chemical properties, in vivo metabolism and toxicological characteristics. CGN-mediated immune activation centers on TLR4, with the MyD88-dependent pathway mediating TNF-α production in specific experimental systems, while the TRIF-dependent NLRP3 inflammasome pathway is involved in IL-1β maturation and release. The administration route dictates the pattern of biological effects: intravenous injection directly activates FXII and platelet CLEC-2 to induce rapid thrombus formation; intraperitoneal injection stimulates macrophages to secrete TNF-α and IL-1β and initiates the inflammation–endothelial injury–coagulation cascade. Under specific experimental model conditions, oral CGN generates low-molecular-weight fragments via the gastrointestinal microbiota, disrupts tight junctions, activates TLR4, and induces colitis. These represent experimental-model-dependent outcomes and are not established consequences of conventional human dietary exposure. The mechanisms outlined above were predominantly determined through the use of non-oral administration, high doses, degraded fragments, or in vitro assays. Even oral studies adopted elevated doses and experimental settings distinct from typical human dietary intake; accordingly, these findings cannot be directly extrapolated to routine human dietary exposure. The published literature on CGN-related inflammation and thrombosis demonstrates that experimental models link experimental outcomes and phenotypic readouts with mechanistic interpretations. Animal models, including paw edema, tail thrombosis, and colitis, built upon these mechanisms recapitulate partial features of inflammation–thrombosis across varying severity grades and are applied for mechanistic investigation and drug-efficacy screening.

The following limitations remain: No co-crystal structure has validated the CGN–TLR4/MD-2 complex, and the binding sites for CGN and TLR4 are only supported by inferred models. The MyD88/TRIF dual signaling branch has only been confirmed in mouse primary peritoneal macrophages and remains unvalidated in intestinal–epithelial or endothelial cell systems. Most existing investigations analyzed TLR4 in isolation and neglected the coordinated or compensatory effects of other pattern-recognition receptors, such as TLR2, TLR6, and scavenger receptors. Systematic comparisons across CGN subtypes, molecular weights, and cell types are inadequate. Direct human-tracer data are lacking for the yield, molecular-weight distribution, and exposure concentration of low-molecular-weight fragments produced by gut-microbiota-mediated degradation. Susceptibility disparities between special-population cohorts have not been verified in large clinical cohorts.

Future research priorities should include resolving the CGN–TLR4/MD-2 complex structure and binding sites; independently validating the MyD88/TRIF dual branch in human intestinal–epithelial cells, endothelial cells and organoids; combining gene-knockout mice, primary cells, human organoids and in vivo oral-exposure models to systematically compare CGN subtypes, molecular weights, and cell types while integrating TLR2/6 and scavenger receptors to clarify receptor-utilization patterns and ligand bias; performing human CGN metabolic tracing to quantify intestinal degradation efficiency, fragment molecular-weight distribution, and systemic absorption; carrying out parallel oral and intraperitoneal control experiments using homologous purified dCGN; building large cohorts of susceptible populations to evaluate risks under dietary exposure; and developing high-purity single-subtype CGN and low-inflammatory derivatives. Such investigations can provide more robust scientific evidence for food-additive safety regulation and natural-drug discovery.

## Figures and Tables

**Table 1:** Key experimental evidence for carrageenan-induced inflammation and thrombosis: a summary by route of administration, subtype, and model system.

Subtype	MW	Dosage/Concentration	Animal Strain/Cell Line	Key Findings	Significance	Ref.	HR
**1. In vivo**							
**1.1. Paw/footpad subcutaneous injection**							
**λ-CGN (Sigma,** **St. Louis, MO, USA** **)**	Unspecified	100 μL of 3% (3 mg/paw), single injection	SD rats (female, 230–280 g)	Peripheral CGN inflammation remotely disrupts blood–brain barrier tight-junction protein assembly via ROS-mediated oxidative stress	Provides cerebrovascular experimental evidence for Section 4’s discussion of “ROS → Vascular endothelial injury → Barrier dysfunction”	[21]	Cat B
**CGN (sodium salt, unspecified κ/ι/λ)**	60–100 kDa	25, 50, 100 μg/paw (0.1 mL)	Wistar rats	First description of biphasic inflammatory response induced by CGN (vascular permeability peak at 1 h; edema peak at 4–5 h)	Establishes foundational understanding of CGN inflammatory kinetics	[22]	Cat B
**CGN (unspecified κ/ι/λ, Sigma)**	Unspecified	Edema of the plantar foot: 500 μg/foot; hyperalgesia: 4.5 mg/foot	SD rats	Revealed spatiotemporal regulatory features of COX-2/PGE_2_ in CGN-provoked inflammation, including dose-dependent effects and positive-feedback regulatory loops	Provides core molecular evidence relevant to Section 7.1 regarding COX-2 as a key mediator in CGN-induced inflammation	[23]	Cat B
**CGN (unspecified κ/ι/λ, Sigma)**	Unspecified	500 μg/paw, single injection	CD1 mice and C57BL/6J mice	Systematically characterized temporal profiles and molecular mechanisms in mouse paw edema model: phase-1 mediated by eNOS + COX-1; phase-2 mediated by iNOS + COX-2	Supplies core evidence supporting model standardization and biphasic inflammatory characteristics (Section 7.1)	[24]	Cat B
**CGN (unspecified κ/ι/λ, Sigma)**	Unspecified	500 μg/paw, single injection	Swiss mice (male, 6–8 weeks)	Assessed oxidative stress markers, iNOS/NO, COX-2/PGE_2_, cytokines and NF-κB signaling; demonstrated anti-inflammatory activity of astragalin	Comprehensive evidence illustrating multi-pathway molecular networks underlying CGN-triggered inflammation	[25]	Cat B
**λ-CGN (TCI America)**	Unspecified	0.5 mg per animal, single injection	*Nymphicus hollandicus*	Established avian local inflammation model for inflammation quantification; CGN injection induces fibrosis and scar formation	Offers unique data describing long-term tissue sequelae following CGN-elicited inflammation	[26]	Cat B
**1.2. Intraperitoneal injection**							
**λ-CGN**	Unspecified	500 μg per animal, single injection	Wistar rats	CGN increased peritoneal leukocyte recruitment (78% neutrophils), IL-1β, IL-6, NO, and MDA	Confirmed intraperitoneal CGN activates macrophage–neutrophil inflammatory cascades	[27]	Cat B
**λ-CGN**	Unspecified	500 μg per animal, single injection	Swiss mice	Elevated peritoneal leukocyte infiltration, NO_2_^−^, MPO; decreased GSH	Demonstrated intraperitoneal CGN triggers inflammatory cascades and oxidative stress	[28]	Cat B
**κ-CGN (Sigma)**	Unspecified	50 mg/per animal, single injection	ICR mice	CGN provoked tail thrombosis; shortened bleeding time; elevated TXB_2_, P-selectin, ET-1, TNF-α, IL-1β, IL-6; histology detected thrombi in tail, lung and liver	Supports the “inflammation → endothelial injury → platelet activation → thrombosis” cascade described in the main text	[29]	Cat B
**κ-CGN(Commercial product)**	Unspecified	50 mg/kg, once daily for 3 consecutive days	SD rats (male)	Altered coagulation parameters (decreased PT/APTT/TT; increased FIB) after intraperitoneal CGN exposure	Direct experimental support for subject of Section 4, “Neutrophil activation → NETs → vascular endothelial injury”	[30]	Cat B
**Chondrus crispus extract, Unspecified**	Unspecified	~10 mg/kg, single injection	BALB/c mice	CGN induces neutrophil extracellular traps (NETs) via a ROS-independent pathway	Direct experimental support for subject of Section 4, “Neutrophil activation → NETs → vascular endothelial injury”	[31]	Cat B
**κ-CGN (Sigma)**	Unspecified	50 mg/kg, single injection	BALB/c mice	Systematically verified multi-organ thrombosis (tail, liver, lung) induced by intraperitoneal κ-CGN	Provides histological evidence relevant to Section 4	[32]	Cat B
**κ-CGN (Sigma)**	Unspecified	100 mg/kg, single injection	BALB/c mice	Linked HMGB1/NF-κB/NLRP3 signaling axis to CGN-driven thrombosis model	Supplies novel mechanistic data for Section 4’s subject, “Inflammation → Coagulation activation”	[33]	Cat B
**κ-CGN (Sigma)**	Unspecified	~50 mg/kg, single injection	Kunming mice (male)	Activated coagulation system (decreased APTT/PT/TT; elevated FIB) and suppressed endogenous anticoagulant system (reduced AT-III, PC)	High-quality dataset supporting Section 5’s topic, “Consumptive coagulopathy”	[34]	Cat B
**κ-CGN (Sigma, Merck)**	~700 kDa	~80 mg/kg, single injection	C57BL/6J wild-type and CLEC-2 knockout mice	First demonstration that CLEC-2 serves as the critical receptor for κ-CGN-mediated platelet activation; reveals direct platelet-activating molecular mechanism	New receptor-level mechanistic evidence for Section 5’s topic, “CGN activates coagulation system → platelet participation”	[35]	Cat B
**CGN (unspecified, Sigma)**	Unspecified	50 mg/kg, single injection	Kunming mice (male)	First report of CGN-triggered gut microbiota dysbiosis: reduced Firmicutes and expanded Muribaculaceae	Provides new mechanistic perspective for systemic biological effects of CGN	[36]	Cat B
**CGN (unspecified, κ/ι/λ)**	Unspecified	~20 mg/kg/d for 10 consecutive days	ICR mice (male)	Intraperitoneal CGN altered gut microbiota profile (increased Firmicutes/Bacteroidetes ratio; reduced community diversity); CGN modulates thrombosis via gut–vascular axis	Supplementary microbiota-derived evidence for “inflammation–thrombosis” cascade discussed in Section 4 and Section 5	[37]	Cat B
**1.3. Tail vein injection**							
**κ-CGN (Sigma, Ekasion)**	Unspecified	1 mg/kg, single injection	SD rats (male)	CGN directly enters circulation, activates coagulation within minutes, induces microvascular thrombosis and tail infarction	This model is widely applied to screen antithrombotic bioactivity of natural products (Section 5)	[38,39,40]	Cat B
**λ-CGN (Rex 7425)**	Unspecified	~36 mg/kg, single injection	CBA mice	Characterized in vivo CGN biodistribution, multi-organ injury (hepatic and cardiac coagulative necrosis), macrophage-mediated clearance and granuloma formation	Classic histological documentation of systemic pathological outcomes following intravenous CGN exposure	[41]	Cat B
**1.4. Oral administration**							
**κ-CGN**	Unspecified	100 mg/kg/d, repeated intragastric administration for 14 days	BALB/c mice	14-day κ-CGN pre-treatment aggravated TNBS-evoked colitis: weight loss, higher mortality, exacerbated colonic inflammation	Suggests CGN may amplify inflammatory injury under pre-existing intestinal inflammatory conditions	[42]	Cat B
**κ-CGN (FITC-labeled)**	Acid hydrolysis	100 mg/kg/d, repeated intragastric administration for 14 days	ICR mice	FITC labeling may alter intrinsic physicochemical features of CGN	Pharmacokinetic study of orally administered FITC-CGN	[43]	Cat B
**Degradable CGN (Glaxo France)**	Unspecified	10% (*w/v*) drinking water, for 10 consecutive days	CF1 mice	Produced hallmark enteritis manifestations: bloody diarrhea, colonic mucosal erosion and hemorrhage	Original landmark study establishing “oral degraded CGN → colitis” animal model	[44]	Cat B
**κ-CGN (Sigma)**	Unspecified	0.1% (*w/w*) added to feed for 10 consecutive days	Adult male zebrafish (Danio rerio)	Intestinal inflammation: elevated goblet-cell count, increased TNF-α, COX-2, IL-1β, IL-8; microbiota dysbiosis (decreased Proteobacteria, increased Fusobacteria)	Cross-species validation evidence for Section 7 “Oral CGN → gut-microbiota-mediated degradation → colitis”	[45]	Cat B
**1.5. Intramuscular injection**							
**λ-CGN (Sigma)**	Unspecified	300 μg/animal, once a week for 6 consecutive weeks	SD rats (male)	Muscle-fiber splitting, inflammatory cellular infiltration; elevated NF-κB, TNF-α, IL-1β, IL-6, LDH, CK; grip strength reduced by 55%	CGN can be utilized to establish experimental acute muscle-inflammation models	[46]	Cat B
**1.6. Air pouch subcutaneous injection**							
**λ-CGN**	Unspecified	1 mg/kg, single injection	Swiss mice (male)	Increased polymorphonuclear leukocytes, monocytes, TNF-α and AnxA1 expression; CGN-provoked inflammation up-regulates endogenous anti-inflammatory AnxA1	New insights into the endogenous resolution mechanisms of CGN-driven inflammation	[47]	Cat B
**2. In vitro**							
**2.1. Intestinal epithelial cells**							
**λ-CGN**	~1000 kDa	1 μg/mL, 24 h	NCM460	CGN engages TLR4; neutralizing antibodies or siRNA knockdown suppress IL-8 secretion >80% in a MyD88-dependent fashion	Defines core molecular inflammatory-activation mechanism triggered by CGN	[48]	Cat C
**λ-CGN**	~1000 kDa	1 μg/mL, 2–24 h	Human Colon Epithelial Cells	Identical TLR4-MyD88-dependent response as NCM460	Primary human-derived cells strengthen human-relevant mechanistic evidence	[48]	Cat C
**κ-CGN**	>1000 kDa	~1 μg/mL, 24 h	NCM460	α-1 → 3-galactoside moiety represents key TLR4-recognised epitope; cleavage of β-1 → 4 glycosidic bonds exposes epitopes and augments pro-inflammatory activity	Elucidates structural-function molecular basis for CGN bioactivity	[49]	Cat C
**κ-CGN (short chain, 8 disaccharide units)**	~4000 Da	~1 μg/mL, 24 h	NCM460	Lower-molecular-weight short-chain κ-CGN exhibits enhanced epitope exposure and stronger intrinsic pro-inflammatory potency	Direct structure–activity experimental evidence supporting “molecular-weight-dependent toxic potential” hypothesis	[49]	Cat C
**κ-, ι-, λ-CGN**	Food-grade	100 μg/mL, 24 h	Caco-2/HT29-MTX	None of three CGN subtypes altered TEER, even under inflammatory priming; no barrier disruption observed up to 10 mg mL^−1^	In vitro data indicate food-grade CGN does not directly disrupt intestinal epithelial barrier function	[50]	Cat C
**κ-CGN**	Food-grade	100 μg/mL, 48 h	Crohn’s-disease-patient-derived colonic organoid monolayers	CGN exerted stronger pro-inflammatory outputs within pre-established inflammatory microenvironments	Indicates potential risk for IBD patients; discrepancies from reference [48] may arise from cell type and disease-state differences	[50]	Cat C
**κ-CGN**	Unspecified	1–10 μg/mL, 24 h	Caco-2 (human colonic epithelium) + THP-1 (PMA-differentiated macrophage-like cells)	Reduced TEER, increased epithelial apoptosis; TNF-α acts as central mediator (blocked by anti-TNFR1 antibody)	Novel mechanism: CGN indirectly impairs intestinal barrier integrity via macrophage-derived TNF-α secretion	[51]	Cat C
**2.2. Macrophages**							
**λ-CGN**	Food-grade	1 μg/mL, 24 h	RAW264.7 mouse macrophages	*Bacteroides xylanisolvens* C3 degrades food-grade λ-CGN into low-molecular-weight fragments	Gut-microbiota-generated CGN degradation fragments possess pro-inflammatory bioactivity	[52]	Cat C
**λ-CGN**	~1000 kDa	1 μg/mL, 24 h	RAW 264.7	CGN binds to TLR4, inducing the secretion of KC (IL-8)	Confirms CGN-TLR4 interaction in macrophages, supporting immune-cell-mediated inflammatory signaling	[48]	Cat C
**λ-CGN**	~1000 kDa	1 μg/mL, 24 h	23ScCr (TLR4 locus deletion)	No CGN binding, no KC secretion	Genetic proof demonstrating TLR4 is indispensable for CGN-triggered responses	[48]	Cat C
**κ-CGN(BDH)**	LMW	300 μg/mL, 6–24 h	Peritoneal macrophages from C57BL/6 and various KO mice	Two independent downstream signaling axes: (1) TLR4/CD14/MyD88 → TNF-α; (2) TLR4/CD14/TRIF/SYK/ROS → pro-IL-1β; NLRP3 inflammasome governs IL-1β maturation	Systematically delineates complete TLR4-dependent and NLRP3-inflammasome-activated molecular cascades within macrophages	[53]	Cat C
**λ-CGN**	Unspecified	30 μg/mL, 3 h	Swiss mouse peritoneal macrophages	Direct CGN macrophage stimulation increased NO_2_^−^ release by 3.2-fold (0.18 → 0.59)	Confirms that CGN can directly activate macrophages to produce inflammatory mediators without auxiliary cell types	[28]	Cat C
**2.3. Whole blood leukocytes plus transfected HEK-293 cells**							
**λ-CGN (Sigma)**	Unspecified	1 μg/mL, 24 h	Yorkshire castrated boar whole blood, HEK-293 cells	λ-CGN activates inflammatory signaling through TLR2/6 and TLR4/6 heterodimers; distinct from LPS-triggered TLR4 homodimer pathway	Provides new mechanistic evidence for TLR-family pattern recognition of CGN	[54]	Cat C
**2.4. Human umbilical vein endothelial cells**							
**κ-, ι-, λ-CGN**	38 × 10^4^ Da; 3.4 × 10^3^ Da	MTT assay: 5–1000 μg/mL, 48 h	Human umbilical vein endothelial cells	Sulfation substitution position (rather than substitution degree) dominates cytotoxic potency; G-6-sulfation is key cytotoxic determinant; A-2-substitution abolishes cytotoxicity	Provides a structural-activity basis for designing low-toxicity CGN derivatives	[8]	Cat C

**Notes:** The Cat A/B/C classification represents an author-defined conceptual framework and is not a formally validated standardized evidence-grading system. The column header “**HR**” stands for “**Human relevance**”, indicating the relevance of the experimental evidence to human food exposure. Relevance to human food exposure is classified as described below. **Cat A:** Studies using food-grade (≥100 kDa) CGN via oral administration at physiologically relevant doses, directly relevant to human food exposure (no such research is cited in this table). **Cat B:** Studies using non-oral routes (i.p., i.v., s.c., etc.), or high doses, or for establishing animal models, which are applicable to understanding the pharmacological/toxicological mechanisms and efficacy screening of CGN but cannot be directly extrapolated to safety evaluation of food additives. **Cat C:** Studies primarily based on in vitro cell experiments, or using non-food-grade components (PGN/LMW fragments), that provide important molecular mechanism hypotheses that require further validation under in vivo oral conditions. **Molecular weight labeling:** “Unspecified” indicates that the original text did not provide molecular weight data; “**~**” indicates an approximate value or range given in the text. Dose conversion explanation: in vivo dose units have been standardized to mg/kg (estimated based on animal body weight), and in vitro units are μg/mL or mg/L. **Abbreviations: AnxA1** (annexin A1); **APTT** (activated partial thromboplastin time); **AT-III** (antithrombin III); **CD** (Crohn‘s disease); **CK** (creatine kinase); **CLEC-2** (C-type lectin-like receptor 2); **COX-1** (cyclooxygenase-1); **COX-2** (cyclooxygenase-2); **eNOS** (endothelial nitric oxide synthase); **ET-1** (endothelin-1); **FIB** (fibrinogen); **FITC** (fluorescein isothiocyanate); **GSH** (reduced glutathione); **IL-1β** (interleukin-1 beta); **IL-6** (interleukin-6); **IL-8** (interleukin-8/CXCL8); **iNOS** (inducible nitric oxide synthase); **i.p.** (intraperitoneal); **i.v.** (intravenous); **LDH** (lactate dehydrogenase); **MDA** (malondialdehyde); **MPO** (myeloperoxidase); **MyD88** (myeloid differentiation primary response 88); **NETs** (neutrophil extracellular traps); **NF-κB** (nuclear factor kappa-B); **NO** (nitric oxide); **NLRP3** (NLR family pyrin domain containing 3); **PC** (protein C); **PGE_2_** (prostaglandin E2); **PGN** (poligeenan); **PT** (prothrombin time); **ROS** (reactive oxygen species); **s.c.** (subcutaneous); **SD** (Sprague Dawley); **TEER** (transepithelial electrical resistance); **TLR4** (Toll-like receptor 4); **TNF-α** (tumor necrosis factor alpha); **TRIF** (TIR-domain-containing adapter-inducing interferon-β); **TNP** (2,4,6-trinitrobenzenesulfonic acid); **TT** (thrombin time); **TXB2** (thromboxane B2).

**Table 2:** Comparison of classification and basic characteristics of poligeenan, degraded carrageenan, and carrageenan.

Substance	Classification	Definition and Preparation	Mw	Ref.
Food-grade CGN	Natural macromolecular sulfated polysaccharide; food additive (E407)	Extracted from red algae (*Chondrus crispus*, *Eucheuma*, etc.) with dilute alkali + alcohol precipitation; non-degraded	≥100 kDa (reported range; typical 200–800 kDa)	[15,60,69]
LMW-CGN/dCGN	Partially degraded product of CGN; non-food grade	Mild degradation of CGN via heat, oxidation, extreme alkali/weak acid	20–40 kDa (reported range)	[15,60,69]
	Food-grade CGN may generate trace amounts of LMW fragments in situ via gut microbiota	Limited degradation of food-grade CGN by gut microbiota (in situ, trace amounts)	Remains uncertain	[52,64]
Poligeenan (PGN)	Completely acid-hydrolyzed product; pharmaceutical/industrial grade, non-food additive	Deep hydrolysis of CGN with dilute acid (pH 0.9–1.5) at 80–95 °C	10–20 kDa (reported range)	[15,60,69]

Molecular weight (Mw) ranges are reported values from the cited studies and are not absolute boundaries applicable to all commercial or experimental materials. LMW-CGN/dCGN as an industrial/experimental preparation is non-food grade; trace fragments generated in situ from food-grade CGN by gut microbiota are not equivalent to industrial LMW-CGN/dCGN. The extent, molecular weight distribution, and biological significance of microbiota-derived fragments in humans under normal dietary exposure remain uncertain.

**Table 3:** In vivo processes and toxic characteristics of different types of carrageenan.

Substance	Intestinal Absorption	Generation and Degradation	Metabolic Excretion	Distribution In Vivo	Intestinal Toxicity	Organ and Immune Toxicity	Carcinogenicity	Safety Assessment	Ref.
Food-grade CGN	In animal studies at normal dietary exposure levels, the majority is excreted in feces as an intact polymer [Animal]. Human data are limited	No consistent evidence of in vivo generation of PGN from intact CGN. In vitro fermentation studies show limited degradation to 40–50 kDa fragments [In vitro/Animal]	In animal studies with intact intestine, the majority is excreted in feces as intact polymer [Animal]	In animal studies under normal dietary exposure, intact food-grade CGN is largely confined to the gastrointestinal tract; systemic distribution is not consistently detected [Animal]. Human data are limited	In animal studies at normal dietary exposure levels, no consistent evidence of intestinal inflammation, ulceration, or epithelial hyperplasia has been observed [Animal]. Human evidence is limited	In animal studies at normal dietary exposure levels, no consistent evidence of hepatic/renal/blood abnormalities or excessive immune activation has been observed [Animal]. Human evidence is limited	No consistent evidence of carcinogenicity at normal dietary exposure levels [Animal/Regulatory]. FDA recognizes GRAS status [Regulatory]	Based on existing normal dietary exposure data, JECFA/EFSA considers that food-grade CGN does not require a specified ADI [Regulatory assessment]	[52,60,66,67,68]
LMW-CGN/dCGN	In experimental studies, minimal absorption (<1%) has been reported, indicating limited intestinal permeability [Animal/Experimental]	Food-grade CGN may naturally contain trace amounts (<5%) of low-molecular-weight fractions [Analytical]. Microbiota-mediated degradation can produce low-molecular-weight fragments, but human in vivo evidence is limited [In vitro/Animal]	Partial intracellular retention with fecal excretion as the main route has been reported in animal studies [Animal]	In animal studies, distribution from gastrointestinal tract to mesenteric lymph nodes and hepatic Kupffer cells has been observed, with low-level distribution in blood and liver [Animal]	In animal studies, high doses (>5%) have been associated with mild intestinal inflammation; no consistent evidence of ulcer formation [Animal]. Human data are limited	In animal studies, accumulation in hepatic Kupffer cells has been observed without consistent evidence of substantial organ damage [Animal]. Human data are limited	No consistent evidence of tumor induction in available studies; long-term human data are lacking [Animal/Human]	Trace (<5%) intake in food has not been consistently associated with clear health hazards in the normal population; evidence is limited [Regulatory/Human]	[52,60,65,67,68]
Poligeenan (PGN)	In animal studies, significant absorption (10–30%) has been reported, indicating efficient intestinal permeability [Animal]	No consistent evidence that intact CGN is converted to PGN in vivo; PGN is artificially prepared by industrial acid hydrolysis [Experimental/Regulatory]	Lysosomal degradation with immune cell involvement has been reported in animal studies [Animal]	In animal studies, after gastrointestinal absorption, PGN distributes to liver, spleen, lymph nodes, and bone marrow, and can cross placental and breast milk barriers [Animal]	In animal studies, PGN induces severe enteritis, ulcers, and polyps [Animal]. Human data are limited	In animal studies, PGN causes hepatosplenomegaly, immune dysfunction, and anemia [Animal]. Human data are limited	Suspected carcinogen based on animal evidence; human evidence is limited [Animal/Regulatory]	In 1972, the FDA prohibited its use in oral preparations, with no food application approval [Regulatory]	[16,19,60,64,67,68]

Notes: “Normal dietary exposure level” refers to conventional dietary intake based on JECFA/EFSA assessments (typically <50 mg/kg/d). “High dose (>5% feed additive)” refers to animal feeding doses exceeding normal dietary exposure levels under experimental conditions, and its conclusions cannot be directly extrapolated to human normal dietary exposure. LMW-CGN/dCGN: low-molecular-weight carrageenan/degraded carrageenan; PGN: poligeenan; GRAS: generally recognized as safe; ADI: acceptable daily intake. [Animal] = animal studies; [Human] = human studies; [In vitro] = in vitro studies; [Regulatory] = regulatory assessments; [Analytical] = analytical chemistry data.

## Data Availability

No new data were created or analyzed in this study. Data sharing is not applicable to this article.
